# Hemolytic disease of the fetus and newborn: systematic literature review of the antenatal landscape

**DOI:** 10.1186/s12884-022-05329-z

**Published:** 2023-01-07

**Authors:** Derek P. de Winter, Allysen Kaminski, May Lee Tjoa, Dick Oepkes

**Affiliations:** 1 Department of Pediatrics, Division of Neonatology, Willem-Alexander Children’s Hospital, Leiden University Medical Center, Leiden, The Netherlands; 2 Department of Immunohematology Diagnostic Services, Sanquin Diagnostic Services, Amsterdam, The Netherlands; 3 OPEN Health, Bethesda, MD USA; 4 Present address: The George Washington University, Washington, DC, USA; 5 Janssen Pharmaceuticals, Raritan, NJ USA; 6 Division of Fetal Medicine, Department of Obstetrics, Leiden University Medical Center, K-06-35, PO Box 9600, Leiden, 2300 RC The Netherlands

**Keywords:** Hemolytic disease of the fetus and newborn, Fetal therapy, Fetal anemia, Intrauterine transfusion

## Abstract

**Background:**

Prevention of pregnancy-related alloimmunization and the management of hemolytic disease of the fetus and newborn (HDFN) has significantly improved over the past decades. Considering improvements in HDFN care, the objectives of this systematic literature review were to assess the prenatal treatment landscape and outcomes of Rh(D)- and K-mediated HDFN in mothers and fetuses, to identify the burden of disease, to identify evidence gaps in the literature, and to provide recommendations for future research.

**Methods:**

We performed a systematic search on MEDLINE, EMBASE and clinicaltrials.gov. Observational studies, trials, modelling studies, systematic reviews of cohort studies, and case reports and series of women and/or their fetus with HDFN caused by Rhesus (Rh)D or Kell alloimmunization. Extracted data included prevalence; treatment patterns; clinical outcomes; treatment efficacy; and mortality.

**Results:**

We identified 2,541 articles. After excluding 2,482 articles and adding 1 article from screening systematic reviews, 60 articles were selected. Most abstracted data were from case reports and case series. Prevalence was 0.047% and 0.006% for Rh(D)- and K-mediated HDFN, respectively. Most commonly reported antenatal treatment was intrauterine transfusion (IUT; median frequency [interquartile range]: 13.0% [7.2–66.0]). Average gestational age at first IUT ranged between 25 and 27 weeks. weeks. This timing is early and carries risks, which were observed in outcomes associated with IUTs. The rate of hydrops fetalis among pregnancies with Rh(D)-mediated HDFN treated with IUT was 14.8% (range, 0–50%) and 39.2% in K-mediated HDFN. Overall mean ± SD fetal mortality rate that was found to be 19.8%±29.4% across 19 studies. Mean gestational age at birth ranged between 34 and 36 weeks.

**Conclusion:**

These findings corroborate the rareness of HDFN and frequently needed intrauterine transfusion with inherent risks, and most births occur at a late preterm gestational age. We identified several evidence gaps providing opportunities for future studies.

**Supplementary Information:**

The online version contains supplementary material available at 10.1186/s12884-022-05329-z.

## Background

Despite advances in the prevention of pregnancy-related red blood cell immunization and management and treatment of pregnancies affected by hemolytic disease of the fetus and newborn (HDFN) over recent decades, the disease still poses a significant risk in affected pregnancies [1, 2]. HDFN is caused by maternal alloimmunization through exposure to incompatible red blood cell antigens of the fetus or through incompatible blood transfusion [1, 3]. The then-formed immunoglobulin G (IgG) antibodies are actively transported across the placenta and can cause fetal hemolysis and anemia. When untreated, progressive fetal anemia results in hydrops fetalis and ultimately fetal demise. If the fetus survives, persistent hemolysis causes neonatal anemia and hyperbilirubinemia, which—when untreated—ultimately leads to a severe cerebral condition (“kernicterus”).

No cure exists for HDFN. Hence, interventions have focused on its prevention and minimizing adverse effects of associated complications [1, 4]. Through transfusing women within the reproductive ages with Kell-negative donor blood, if possible, and through the introduction of Rhesus (Rh) immunoglobulin prophylaxis, the occurrence of red blood cell alloimmunization and the prevalence of Rh(D)- and K-mediated HDFN has decreased [1, 4–6]; however, the gap between anti-Rh(D) supply and demand is large in low-income countries and is below the optimal threshold in high-income countries [7]. Additionally, the disease still poses a significant risk for mortality and morbidity in developing countries, whereas it is considered treatable with good outcomes in developed countries. Serological monitoring, ultrasonography, and Doppler imaging decreased the need for risky and invasive diagnostic procedures [3, 8–12]. Antenatal treatment, however, still relies predominantly on (often serial) intrauterine transfusion (IUT)—an invasive procedure that carries maternal and fetal risks [13, 14].

Considering improvements in HDFN care, the objectives of this systematic literature review were to assess the prenatal treatment landscape and outcomes of Rh(D)- and K-mediated HDFN in mothers and fetuses to identify the burden of disease, to identify evidence gaps in the literature, and to provide recommendations for future research. Secondarily, we aim to determine the humanistic and economic burden of HDFN.

## Methods

### Search strategy

**Fig. 1:**
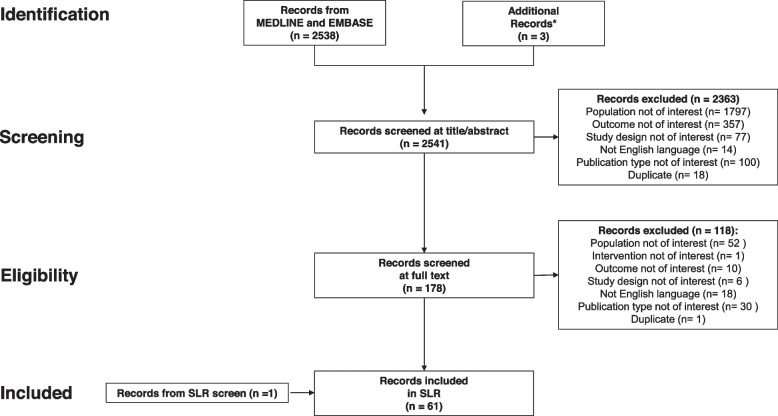
Flowchart of the Article Selection Process. SLR, systematic literature review. ^*^From authors’ personal library. ^†^From eligible SLRs of cohort studies

We conducted a systematic literature review according to the Preferred Reporting Items for Systematic Reviews and Meta-Analyses (PRISMA) statement [15] and MOOSE Reporting Guidelines for Meta-Analysis of Observational Studies [16] to address prespecified research questions (Table S2). To assess the treatment landscape, articles published between January 1, 2005, and March 10, 2021 were searched (Additional file 2: Appendix S1) in the MEDLINE and EMBASE databases and ClinicalTrials.gov using ProQuest (Fig. 1). The search strategy included descriptions of the disease, possible interventions and clinical outcomes. No limitations were set on studies reporting on cases managed before January 1, 2005. Searches for clinical outcomes were performed for journal articles and conference abstracts indexed in EMBASE. Duplicates were removed automatically. We also manually searched reference lists of pertinent systematic literature reviews of cohort studies and our personal libraries for potentially relevant articles.

### Study selection

Two independent reviewers (D.P.D.W. and A.K.) (Table S3) [17] reviewed the titles/abstracts in Rayyan (https://rayyan.ai/) and then full texts in Microsoft Excel. Citations were independently evaluated to determine whether or not studies fulfilled inclusion and exclusion criteria. The project director (D.O.) and the project team adjudicated decisions. Randomized or nonrandomized trials; retrospective or prospective observational studies, including cohort, case-control, or cross-sectional studies; modelling studies; systematic reviews of cohort studies (to identify primary studies only); and case reports and case series of women and/or their fetuses, infants, or children experiencing or having experienced Rh(D)- and/or K-mediated HDFN were included. Studies or patient groups within studies where HDFN was caused by alloimmunization to antigens other than Rh(D) only and/or K only, such as c, e, E, Duffy (Fy), Kidd (Jk), MNS (S), or Gerbich, were excluded as the risk of prenatal disease is regarded as relatively low. Non–English-language articles were excluded, as were notes, editorials, and commentaries; nonsystematic reviews; reports of populations, interventions, outcomes, or study designs not of interest; publication types not of interest; indexed conference abstracts; and reports of animal or preclinical studies. The review was registered with PROSPERO before data were abstracted.

Two independent reviewers (D.P.D.W. and A.K.) abstracted data (i.e., study reference; study design; patient characteristics; HDFN treatment patterns; clinical outcomes [eg, fetal anemia, hydrops fetalis, and adverse events]; intravenous immunoglobulin [IVIG] efficacy; mortality; and prevalence) from studies that fulfilled inclusion and exclusion criteria. All abstracted data underwent quality control by the project director (D.O.), who screened 10% of included/excluded articles. The methodological quality (risk of bias) of the selected studies was assessed by 2 independent reviewers (D.P.D.W. and A.K.) using the JBI Critical Appraisal Checklist for Case Reports [18], the JBI Critical Appraisal Checklist for Case Series [19], the Newcastle-Ottawa Scale for retrospective and prospective cohort studies [20], the Checklist for Reporting Results of Internet E-Surveys (CHERRIES) for questionnaires [21], and lastly the NICE checklist for randomized controlled trials (RCTs).

### Analyses

Data from eligible studies were characterized as representative, which included data from studies that accurately reflected the characteristics of the larger group (e.g., larger case series, retrospective or prospective studies, RCTs), or were characterized as nonrepresentative, which included data from studies that reflected a small proportion of the characteristics of the larger group (e.g., case reports or small case series, or studies in a subset of the larger group, such as cases treated with IUT or only cases with hydrops fetalis). When possible, we aggregated information reported in a similar manner. For unique outcomes, we highlighted information from generalizable studies. Where appropriate, data were summarized as percentage (mean ± standard deviation [SD] or range) or median (interquartile range [IQR]) for patient groups or patient populations (e.g., Rh[D] or Kell, Rh[D] treated with IVIG or Rh[D] not treated with IVIG). Case reports and case series were excluded from prevalence analyses.

An assessment of the available findings was conducted to identify evidence gaps, and recommendations to fill unmet needs were formulated. Results pertinent to mothers and fetuses are reported herein. Neonatal outcomes will be reported in a separate article.

### Humanistic and economic burden

A separate objective of this systematic review is to determine the humanistic and economic burden of HDFN. We conducted a systematic search using the same criteria as previously mentioned (Additional file 2: Appendix S2). We selected studies reporting on quality of life, humanistic burden, economic burden, health care resource use, and direct and indirect costs. The review process was performed according to the PRISMA and MOOSE guidelines and similar as previously stated.

## Results

### Data sources

In addition to the 2,538 articles identified through searches of MEDLINE and EMBASE, we identified 3 articles from our personal libraries (Fig. 1). The search on ClinicalTrials.gov did not yield any additional results to the search on MEDLINE and EMBASE. Overall, 2,363 of the 2,541 total articles were excluded on the basis of title and abstract review, and 119 were excluded on the basis of full-text review. Besides the 59 articles that remained, we identified 1 article from a review of applicable systematic reviews of cohort studies.

### Study characteristics

Among the 60 eligible studies that were included in our analysis (Table S1), [2, 22–80] nearly half were retrospective cohort studies (*n* = 27 [45%]), followed by case reports and case series (*n* = 21 [35%]), prospective cohort studies (*n* = 7 [12%]), observational cohort studies (*n* = 3 [5%]), and RCTs and questionnaires (*n* = 1 [2%] each). More studies included patients with only Rh(D)-mediated HDFN (*n* = 26) than only K-mediated HDFN (*n* = 7); 27 studies included patients with Rh(D)- or K-mediated HDFN. Studies were conducted across 25 countries, most commonly The Netherlands (*n* = 12), followed by Turkey and the United States (*n* = 6 each). The 60 studies comprised 146 patient groups, including mothers, neonates, and fetuses. Of these patient groups, 46 were single patients extracted from case reports. Mean (range) group size, including case reports, was 36.5 (SD ± 68.7, range 1.0–334.0). The reported patient groups included cases managed between 1985 and 2019.

### Methodological quality of the studies

Of the 14 included case reports, 7 received a perfect score (8/8) using the JBI Critical Appraisal Checklist for Case Reports. Median score among case reports was 7.5 [IQR: 6.0–8.0]. Two of the seven included case series received a perfect score among the applicable questions. Median score among case series was 5.0 [IQR: 5.0–8.5]. Twenty-one of 30 retrospective cohort studies were rated as good quality, 8 as fair, and 1 as poor. All 8 included prospective studies were rated as good quality. The randomized controlled trial by Santos et al. was rated as having low risk of bias in all 4 domains—selection bias, performance bias, attrition bias, and detection bias. Tables S4a-e contains a detailed overview of the methodological quality of the selected studies.

### Diagnostic testing

Diagnostic testing data were available for 59 of the 60 studies. The most commonly reported diagnostic testing method was ultrasound (*n* = 20; median [IQR]: 100% [100–100%]), followed by percutaneous umbilical cord sampling (*n* = 17; 100% [100–100%]); anti-D and/or anti-K antibody titer (*n* = 16; 100% [100–100%]); fetal hemoglobin (*n* = 15; 100% [100–100%]); Coombs/antiglobulin testing (*n* = 15; 100%); cell-free DNA testing (*n* = 8; 100% [80–100%]); amniocentesis (*n* = 7; 100% [50–100%]); free antibody testing, antibody release testing, and gel card technique (*n* = 2; 100%); and magnetic resonance imaging (*n* = 2; 100% [56.5–100%]).

### Prevalence of D- and K-mediated HDFN

**Fig. 2:**
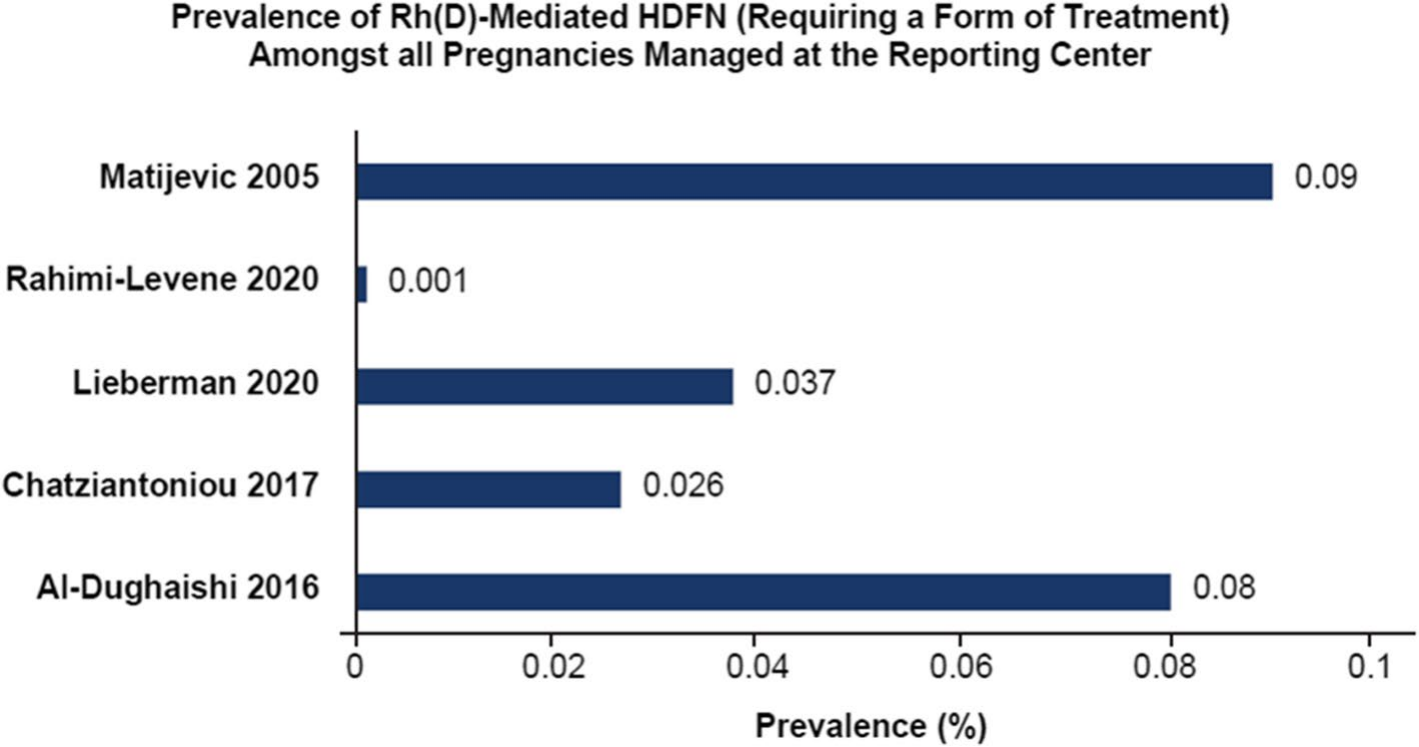
Prevalence of Rh(D)-mediated HDFN (Requiring a Form of Treatment) Among All Referred Pregnancies [18, 24, 40, 42, 49]. D, Rh(D); HDFN, hemolytic disease of the fetus and newborn; Rh, Rhesus; SD, standard deviation

The mean ± SD prevalence of Rh(D)-mediated HDFN (requiring any form of treatment) as reported in 5 studies [23, 29, 45, 47, 54] was 0.047%±0.037% among all pregnancies that were managed and delivered in the centers of the 5 selected studies (Fig. 2). The reported prevalence of K-mediated HDFN (requiring any form of treatment) among all pregnancies managed and delivered in the centers reporting in 2 retrospective studies was 0.006% [45, 47]. No data were available for the prevalence of early-onset HDFN (requiring intervention before 24 weeks of pregnancy) in the selected studies. The gestational age at first IUT was between 25 and 27 weeksand the mean gestational age at birth between 34 and 36 weeks (Table 1) amongst all selected studies [2, 22–80].

**Table 1:** Patient Characteristics Among 155 HDFN Groups, Including Mothers, Neonates, and Fetuses, From 60 Included Studis

Characteristic	Mean (± SD, Range)	Patient Groups (n)	No. of Studies (References)
Patient group size, n	36.5 (± 68.7, 1.0–334.0)	146^a^	60 [2, 22–80]
Gestational age at birth, weeks^b^			
Mean gestational age	35.1 (± 2.1, 33.0–37.4)	13	9 [23, 26, 28, 47, 53, 63, 65, 70, 72]
Median gestational age	35.4 (± 2.1, 28.0–37.0)	24	12 [31, 35, 42, 45, 51, 59, 60, 62, 63, 75, 76]
Exact gestational age	34.1 (± 2.9, 28.1–38.0)	18	14 [22, 24, 27, 34, 39, 43, 44, 46, 50, 52, 55, 66, 74, 77]
Gestational age at first IUT, weeks			
Mean gestational age	26.6 (± 0.1, 26.6–26.7)	4	2 [28, 53]
Median gestational age	25.9 (± 3.4, 21.7–36.3)	27	13 [31, 51, 59–61, 63, 64, 69, 73, 75, 79, 80]
Exact gestational age	24.8 (± 3.9, 20.0–30.1)	8	5 [44, 52, 66, 68, 76]

Means, medians, and exact gestational age as reported in each study were used to calculate mean (range)

*HDFN* Hemolytic disease of the fetus and newborn, *IUT* Intrauterine transfusion

^a^Of the 155 patient groups, 46 (29.7%) groups were single patients from case reports

^b^Does not include 1 study [2] in which the reported percentage of the patient group fell within gestational age ranges (i.e., < 259 days and 259–294 days)

### Frequencies of antenatal management strategies

Antenatal treatment data were available for 24 studies (Table S8) [24, 25, 29, 30, 33–37, 42, 43, 48, 51, 52, 56, 58–63, 65, 71, 80]. The most commonly reported antenatal treatment across these studies was IUT (*n* = 9 with representative data [25, 29, 33, 36, 37, 56, 59, 60, 65]; *n* = 3 with nonrepresentative data [35, 58, 62].

### Intrauterine transfusions

Among the 9 studies with representative data, IUTs were given at a median frequency of 13.0% (IQR: 7.2–66.0) among pregnancies with a positive anti-Rh(D) screening that were monitored prenatally with ultrasonography. Three of these studies monitored pregnancies with a positive anti-Rh(D) screening and a risk-stratification using the antibody titers and/or antibody-dependent cellular cytotoxicity values above cut-off value, and might therefore overestimate the frequency of IUTs [56, 59, 60]. In these three studies, the frequency of IUTs was 64.7% (range 59.2–66) [56, 59, 60]. Number of IUTs was reported by only two of these studies with a median of 2 (range 0–4) [56, 59]. IUTs were required in 76.8% of pregnancies, as reported in 63/82 collective cases [36, 56, 60].

The frequency of IUTs in pregnancies with a positive anti-Rh(D) screening monitored without serological cut-off values was 11.2% (range, 4.5–58.6) in the collective cases (42/376) reported by 5 studies [25, 29, 33, 37, 65]. The number of IUTs required was only reported by 1 study with a mean of 2.4 (SD not reported) [33]. Data on the need for IUTs in pregnancies with K-alloimmunization without serological cut-offs were reported by 1 study and was 12.5% in 1/8 reported cases [25].

### Alternative management strategies

Use of IVIG alone was reported in 1 study with representative data [48]. In this case series of 3 severely affected pregnancies, 2 (Rh[D], *n* = 1; Kell, n = 1) resulted in live births without IUT; the third (Rh[D] + anti-C) was treated with IUT but resulted in a post-procedure intrauterine death.

Use of IUT + IVIG was reported in 4 studies (*n* = 1 with representative data[61]; *n* = 3 with nonrepresentative data [35, 42, 62]). In the one study with representative data, 3.2% of the Rh(D) alloimmunization cases and 4.3% of the Kell alloimmunization cases were treated with IUT + IVIG [61].

Use of other treatments (therapeutic plasma exchange [TPE]; maternal plasma exchange ± high-dose IVIG; TPE + IVIG + IUT; TPE + immunoadsorption + IVIG + IUT; plasmapheresis + IUT; and plasmapheresis + IVIG + IUT) were reported in 10 studies with a total of 38 cases [24, 30, 34, 36, 43, 51, 52, 63, 71, 80]. Plasmapheresis + IVIG + IUT was the most commonly reported treatment regimen across these 10 studies.Two of these studies did not report gestational age at start of the treatment, at first IUT (if applicable) and at birth [36, 80]. The remaining 8 studies, including 20 cases total, reported the mean gestational age at treatment initiation (13.0 ± 5.7 weeks). 17/20 cases required an IUT for fetal anemia. The mean gestational age at first IUT was 24.2 ± 3.1 weeks, with a median of 4 IUTs (range 1–8) administered. Gestational age at birth was 34.4 ± 3.1 weeks [30, 34, 43, 51, 52, 63, 71]. In the series of 20 cases, one patient received plasmapheresis, which was started a week after the first IUT at a gestational age of 27 weeks [43]. In all other cases, the alternative treatment was started prior to the occurrence of fetal anemia. The indications to start the alternative treatment option in the 20 cases were previous intrauterine fetal death (*n* = 11), neonatal hydrops fetalis and/or death (*n* = 4), marked elevation in antibody titer (*n* = 4), and suspected fetal anemia after initial IUT (*n* = 1).

### Clinical outcomes of mothers and fetuses

The most commonly reported maternal/fetal clinical outcome across studies was hydrops fetalis (*n* = 19 with representative data [23, 28, 31, 32, 41, 42, 47, 49, 51, 53, 59, 64, 66, 67, 69, 75, 76, 79, 80] (Table S8); *n* = 10 with nonrepresentative data [24, 39, 40, 44, 46, 52, 55, 68, 70, 74]). The rate of hydrops fetalis among pregnancies with Rh(D)-mediated HDFN treated with IUT was 14.9% (range, 0–50%) in 72/483 reported cases [31, 32, 49, 53, 66, 76, 79]. The rate of hydrops fetalis among pregnancies with K-mediated HDFN treated with IUT was 39.2% in 49/125 reported cases [69, 75, 76, 79]. Five studies reported on the rate of hydrops fetalis in all pregnancies monitored for Rh(D)- and or K-alloimmunization, with or without the need for antenatal treatment. The rate of hydrops fetalis in these studies was 7.3% in 17/232 collective cases.

Severe fetal anemia was reported in 1 study with representative data [28] (Table S8) and 11 studies with nonrepresentative data [30, 39, 42–44, 46, 52, 63, 68, 74, 80]. In the 1 cohort study with representative data, 100% of 22 successful IUTs performed in Rh(D)- or K-mediated HDFN cases within 20 weeks of gestation were considered severely anemic (≥ 5 SDs from the fetal hemoglobin reference value of 15 g/dL; 1 SD = 1 g/dL difference from reference value) [28]. Adverse events or procedure-related complications were commonly reported after IUTs or other treatments for Rh(D)- and/or K-mediated HDFN (*n* = 11 studies with representative data [28, 33, 47, 51, 53, 63, 64, 66, 69, 72, 80] (Table S8); *n* = 2 studies with nonrepresentative data [68, 73]). Bradycardia was the most frequently reported post-IUT complication per procedure, and adverse serological outcomes were the most frequently reported post-IUT complication per fetus, although adverse serological outcomes were reported in only 1 study [33] (Fig. S2).

### IVIG efficacy

Assessment of IVIG efficacy in women and fetuses affected by HDFN was based on treatment response in 6 studies [24, 30, 34, 48, 62, 80] and associated mortality in 3 studies [35, 42, 63] (Table S8). Collectively, findings indicate that IVIG delayed or prevented IUT. IVIG-associated fetal mortality ranged from 0 to 50% across the 3 studies reporting this outcome [35, 42, 63].

### Fetal mortality

The overall mean ± SD fetal mortality rate was 19.8% ± 29.4% across 19 studies, including representative case reports [28, 31, 35, 42, 43, 45, 47, 49, 53, 63, 64, 66, 68, 69, 73, 75, 76, 78, 80] (Table S8). Head-to-head comparison of mortality rates between the different treatment strategies is limited by variation in potential patient characteristics between the groups. Employed treatment strategies for HDFN take into account previous obstetrical history, for example 75% of cases in the “IUT + other” group had a history of fetal or neonatal death due to HDFN. Together, these will influence the outcomes of HDFN in the current pregnancy and limit our capability of mortality rate comparison.

### Humanistic and economic burden

In addition to the 1457 articles identified in the systematic search, one additional article was identified from personal libraries (Fig. S2). Based on the title/abstract screening 1435 articles were excluded. Full-text screening was performed in the remaining 23 records of which 22 were excluded. Healthcare utilization was reported by only one study with a median of duration of phototherapy of 4-4.5 days and a median length of stay of 6.5–7.5 days [65].

## Discussion

### Main findings

We found that the prenatal burden and need for treatment remains relatively high – we estimated that 13% of pregnancies monitored for Rh(D) or K-alloimmunization required one or more IUTs – despite advances in the identification and care for pregnancies at risk of HDFN. Strikingly, the rate of hydrops fetalis in pregnancies requiring an IUT was found to be 14.9% for Rh(D)-mediated HDFN and 39.2% in K-mediated HDFN. As the occurrence of hydrops fetalis was previously found to be associated with impaired neurodevelopmental outcomes [81] still much is to gain in the timely identification of pregnancies at-risk and the timely detection and treatment of fetal anemia to prevent hydrops fetalis. The average gestational age at first IUT was 27 weeks, which was possibly delayed by using IVIG and/or plasmapheresis although evidence on this in the included studies is limited. Although IUT is a regarded as a relatively safe procedure in experienced hands, its invasive nature still poses serious risks to the mother and fetus. Fetal loss rate increases when procedures need to be done early in gestation (i.e., < 22 weeks) [13]. It is also noteworthy that the average gestational age at birth in the present analyses was approximately 35 weeks for Rh(D)- and/or K-mediated HDFN, which is considered late preterm and might also represent that early delivery is frequently employed in the management of pregnancies at risk of fetal anemia although we were unable to extract data on this from the included studies. But, late preterm birth has the potential for serious consequences, such as increased risk for short- or long-term respiratory issues [82–84], readmission [82], death [82, 84, 85], and neurocognitive impairment in late adulthood [86, 87].

### Strengths and limitations

A strength of this systematic review and corresponding analyses is the minimal limitation on study design criteria. Overall, 35% of the studies included in our analyses were case reports or case series, validating the rarity of HDFN. By including case reports and case series, we were able to identify and aggregate data on treatment types (e.g., plasmapheresis and plasma exchange) that were not typically reported in larger cohort studies. But, inclusion of case reports and case series might also be regarded as a limitation as it may skew and overestimate the results as, given the rarity of HDFN, the most severe cases are generally reported in literature. Our estimates might therefore not truly mirror the population level data.

Also, we were unable to quantify heterogeneity (using e.g. the I^2^-statistic) due to the descriptive nature of this systematic review and consequent lack of reported comparisons between interventions. However, a certain level of heterogeneity may be expected due to differences in available management options, treatment protocols, prevalence of Rh(D)- and K-negativity, geographical location and sociodemographic differences. These differences may be approached by the varying frequencies of, for instance, IUTs and rate of hydrops fetalis between included studies.

Our analyses are further limited by the strict prespecified inclusion of only Rh(D)- or K-mediated HDFN populations. By applying this criterion, 7 studies were excluded from analyses—despite high quality of evidence and outcomes of interest—because Rh(c) populations were mixed with Rh(D) or Kell populations, or the population was not well defined [88–94]. We were unable to stratify the data per alloimmunization type with the data provided in these articles. One of these studies represented the only prospective study with long-term outcomes [89], thereby also indicating the lack of data on long-term outcomes and the need for further research on the topic.

### Interpretation

Almost all studies included in these analyses were conducted in high-income countries, which have adequate resources for screening, prophylaxis and preventative measures for alloimmunization, and referral to specialized fetal therapy centers. Outcome data on HDFN-complicated pregnancies from less privileged or less organized societies are lacking and, if analyzed, likely are less favorable. This well-known bias in outcome reporting indicates an important evidence gap and signifies the need for international collaboration to gain a better understanding of the global burden of HDFN and to pave the way for potential wide-spread improvements. To add to that, we also found that the evidence for frequency of use and effectiveness of alternative treatment options such as IVIG, plasmapheresis, and plasma exchange on disease severity and the prevention of fetal anemia is limited in the included studies. Also, as previously mentioned it is likely that the most severe cases are reported in literature due to the rarity of the disease. Taken together, future research should aim to gain more exact insight into the employed treatment options and its efficacy and clinical outcomes of mothers, fetuses, and neonates affected by HDFN through an international retrospective and/or prospective registry through the collection of data on diagnostics, antenatal and postnatal treatments and short- and long-term clinical outcomes of mothers, fetuses, and neonates. Such an international effort will pave the way for long sought after answers.

A separate objective of this systematic review, as previously mentioned, was to ascertain the economic and humanistic burden of HDFN. However, through the systematic approach only one study reporting on healthcare utilization was included. This dearth of information indicates another major gap in knowledge, particularly as it relates to the impact of HDFN on a pregnant individual’s quality of life and the potential downstream consequences of high-risk pregnancy on family planning decisions, as well as on the healthcare system.

## Conclusion

To conclude, we found that the clinical burden of Rh(D)- and K-mediated HDFN remains relatively high, with 13% of pregnancies monitored for Rh(D)- or K-alloimmunization requiring an IUT and most births occurring at a late preterm gestational age. We identified several important evidence gaps that provide opportunities for future studies to further improve the clinical care of HDFN.

## Supplementary Information

**Additional file 1: Table S1.** General Characteristics of Studies and Patient Groups. **Table S2.** Research Questions Relevant to Mothers and Fetuses. **Table S3.** Inclusion and Exclusion Criteria^a^. **Table S4a.** Methodologic Quality of Selected Case Reports. **Table S4b.** Methodologic Quality of Selected Case Series. **Table S4c.** Methodologic Quality of Selected Retrospective Cohort Studies. **Table S4d.** Methodologic Quality of Selected Prospective Cohort Studies. **Table S4e.** Methodologic Quality of Bennardello et al (25). **Table S5.** Antenatal Treatments Reported in Studies With Representative Data. **Table S6.** Hydrops Fetalis, Severe Fetal Anemia, and Adverse Events/Complications Reported in Studies With Representative Data. **Table S7.** IVIG Treatment Response and Associated Mortality. **Table S8.** Overall Fetal Mortality Associated With HDFN. **Figure S1.** Mean Rate of Procedure-related Complications After IUT (Per Procedure [A] and Per Fetus [B]).^12,26,30,32,43,44,46,49,52,60^ CS, cesarean section; IUT, intrauterine transfusion; PROM, preterm rupture of membranes. ^a^Adverse serological outcome was defined as the development of additional maternal antibodies to anti-D and/or a ≥4-fold enhancement of antibody titer.^12^. **Figure S2.** Flowchart of the Article Selection Process for the Humanistic and Economic Burden. SLR, systematic literature review. *From authors’ personal library. †From eligible SLRs of cohort studies.

**Additional file 2: Appendix S1.** Search Strategy. **Appendix S2.** Search Strategy for the Humanistic and Economic Burden. 

## Data Availability

The datasets used and/or analysed during the current study available from the corresponding author on reasonable request.

## Abbreviations

HDFN: Hemolytic disease of the fetus and newborn
CHERRIES: Checklist for Reporting Results of Internet E-Surveys
DNA: Deoxyribonucleic acid
IQR: Interquartile range
IUT: Intrauterine transfusion
IVIG: Intravenous immunoglobulins
JBI: Joanna Briggs Institute
MOOSE: Meta-analysis of Observational Studies in Epidemiology
PRISMA: Preferred Reporting Items for Systematic Reviews and Meta-Analyses
RCT: Randomized controlled trial
Rh: Rhesus
SD: Standard deviation
TPE: Therapeutic plasma exchange
USA: United States of America
