# Supplementary material for: Hemolytic disease of the fetus and newborn: systematic literature review of the antenatal landscape
Source: BMC Pregnancy Childbirth. 2023 Jan 7;23:12. doi: 10.1186/s12884-022-05329-z (PMC9824959; doi:10.1186/s12884-022-05329-z)
Supplement: Supplementary file 1 — Additional file 1: Table S1. General Characteristics of Studies and Patient Groups. Table S2. Research Questions Relevant to Mothers and Fetuses. Table S3. Inclusion and Exclusion Criteriaa. Table S4a. Methodologic Quality of Selected Case Reports. Table S4b. Methodologic Quality of Selected Case Series. Table S4c. Methodologic Quality of Selected Retrospective Cohort Studies. Table S4d. Methodologic Quality of Selected Prospective Cohort Studies. Table S4e. Methodologic Quality of Bennardello et al (25). Table S5. Antenatal Treatments Reported in Studies With Representative Data. Table S6. Hydrops Fetalis, Severe Fetal Anemia, and Adverse Events/Complications Reported in Studies With Representative Data. Table S7. IVIG Treatment Response and Associated Mortality. Table S8. Overall Fetal Mortality Associated With HDFN. Figure S1. Mean Rate of Procedure-related Complications After IUT (Per Procedure [A] and Per Fetus [B]).12,26,30,32,43,44,46,49,52,60 CS, cesarean section; IUT, intrauterine transfusion; PROM, preterm rupture of membranes. aAdverse serological outcome was defined as the development of additional maternal antibodies to anti-D and/or a ≥4-fold enhancement of antibody titer.12. Figure S2. Flowchart of the Article Selection Process for the Humanistic and Economic Burden. SLR, systematic literature review. *From authors’ personal library. †From eligible SLRs of cohort studies. [file 12884_2022_5329_MOESM1_ESM.docx]

**SUPPLEMENTAL TABLES**

**Table S1.** General Characteristics of Studies and Patient Groups

| Study | Study Type | Sample Size | Groups | Group Size | Data Collection Period | Country |
| --- | --- | --- | --- | --- | --- | --- |
| Akdag 2012^22^ | Case report | 1 | Neonate, K-mediated HDFN | 1 | NR | Turkey |
| Al-Dughaishi 2016^23^ | Retrospective cohort | 1,251 | Pregnant women, D-mediated HDFN  Pregnant women, K-mediated HDFN | 8  2 | June 2011–June 2013 | Oman |
| Bek 2019^24^ | Case report | 1 | Pregnant women, D-mediated HDFN | 1 | NR | Turkey |
| Bennardello 2013^25^ | Questionnaire | 1,661 | Deliveries (Rh[D]- women), D-mediated HDFN | 111 | NR | Italy |
|  |  |  | Deliveries, K-mediated HDFN | 8 |  |  |
|  |  |  | Neonates, D-mediated HDFN | 111 |  |  |
| Bi 2019^26^ | Prospective cohort | 18 | Neonates, D-mediated HDFN | 5 | January 2017–June 2019 | PRC |
| Brumbaugh 2011^27^ | Case report | 1 | Neonate, K-mediated HDFN | 1 | NR | USA |
| Canlorbe 2011^28^ | Retrospective cohort | 18 | Pregnancies <20 weeks’ gestation treated with IUT | 18 | January 1, 1990–August 15, 2011 | France |
|  |  | 25 | IUTs performed/attempted <20 weeks’ gestation | 25 |  |  |
|  |  |  | IUTs performed/attempted <20 weeks’ gestation, D-mediated HDFN  IUTs performed/attempted <20 weeks’ gestation, K-mediated HDFN | 15  1 |  |  |
| Chatziantoniou 2017^29^ | Retrospective cohort | 130 | Pregnancies, D-mediated HDFN | 32 | June 2006–June 2013 | United Kingdom |
|  |  |  | Neonates, D-mediated HDFN | 22 |  |  |
|  |  |  | Neonate, K-mediated HDFN | 1 |  |  |
| Colpo 2017^30^ | Case report | 1 | Pregnancy, D-mediated HDFN | 1 | NR | Italy |
|  |  |  | Neonate, D-mediated HDFN | 1 |  |  |
| Craparo 2005^31^ | Prospective cohort | 31 | Pregnancies, D-mediated HDFN treated with IUT | 31 | 1998–2002 | Italy |
| De Assuncão 2016^32^ | Prospective cohort | 13 | Pregnancies, D-mediated HDFN treated with IUT | 13 | NR | Brazil |
|  |  |  | Neonates, D-mediated HDFN | 13 |  |  |
| Dubey 2016^33^ | Prospective cohort | 29 | Pregnancies, D-mediated HDFN treated with IUT | 31 | NR | India |
| Fernandez Alba 2014^34^ | Case report | 1 | Pregnancy, D-mediated HDFN | 1 | NR | Spain |
|  |  |  | Neonate, D-mediated HDFN | 1 |  |  |
| Fox 2008^35^ | Case series | 6 | Pregnancies (D- or K-mediated HDFN) in whom in a previous pregnancy the fetus had been hydropic and severely anemic Pregnancies <20 weeks’ gestation associated with a high perinatal mortality | 6 | 1997–2004 | United Kingdom |
|  |  |  | Pregnancies treated with IUT only | 4 |  |  |
|  |  |  | Pregnancies treated with maternal IVIG and IUT | 2 |  |  |
| Gottvall 2008^36^ | Retrospective cohort | 78,145 | Pregnancies, D-mediated HDFN | 71 | January 1992–December 2005 | Sweden |
|  |  |  | Neonates, D-mediated HDFN | 71 |  |  |
|  |  |  | Pregnancies, K-mediated HDFN | 9 |  |  |
| Gudlaugsson 2020^37^ | Retrospective cohort | 132 | Deliveries (Rh[D]- women), D-mediated HDFN | 130 | 1996–2015 | Iceland |
|  |  |  | Fetuses, D-mediated HDFN | 112 |  |  |
|  |  |  |  |  |  |  |
| Haider 2020^38^ | Case report | 1 | Neonate | 1 | NA | Pakistan |
| Harper 2006^39^ | Prospective cohort | 18 | Hydropic fetus, D-mediated HDFN (case #3) | 1 | July 1985–October 1995 | USA |
|  |  |  | Hydropic fetus, K-mediated HDFN (case #7) | 1 |  |  |
|  |  |  | Hydropic fetus, D-mediated HDFN (case #10) | 1 |  |  |
|  |  |  | Hydropic fetus, K-mediated HDFN (case #13) | 1 |  |  |
|  |  |  | Hydropic fetus, D-mediated HDFN (case #15) | 1 |  |  |
| Hassan 2019^40^ | Case report | 1 | Neonate, D-mediated HDFN | 1 | NA | Malaysia |
| Karagol 2012^41^ | Retrospective cohort | 106 | Neonates, K-mediated HDFN | 5 | January 2005–December 2010 | Turkey |
| Kriplani 2007^42^ | Case series | 4 | Pregnancies, D-mediated HDFN | 4 | NR | India |
|  |  |  | Neonates, D-mediated HDFN | 4 |  |  |
| Lakhwani 2011^43^ | Case report | 1 | Pregnancy, K-mediated HDFN | 1 | NR | Spain |
|  |  |  | Neonate, K-mediated HDFN | 1 |  |  |
| Levy-Zauberman 2011^44^ | Case report | 1 | Pregnancy, D-mediated HDFN | 1 | NR | France |
|  |  |  | Neonate, D-mediated HDFN | 1 |  |  |
| Lieberman 2020^45^ | Retrospective cohort | 128 | Mothers, D-mediated HDFN  Mothers, K-mediated HDFN | 16  2 | November 2010–June 2017 | Canada |
|  |  |  | Neonates, D-mediated HDFN  Neonates, K-mediated HDFN | 18  2 |  |  |
| Manoura 2007^46^ | Case report | 1 | Pregnancy, K-mediated HDFN | 1 | NR | Greece |
|  |  |  | Neonate, K-mediated HDFN | 1 |  |  |
| Matijevic 2005^47^ | Retrospective cohort | 23 | Pregnancies, D-mediated HDFN  Pregnancies, K-mediated HDFN | 15  1 | January 1997–January 2003 | Croatia |
| Mayer 2018^48^ | Case series | 3 | Pregnancies, D- or K-mediated HDFN | 2 | NR | Germany |
|  |  |  | Neonates, D- or K-mediated HDFN | 2 |  |  |
| Meraj 2015^49^ | Retrospective cohort | 8 | Fetuses, D-mediated HDFN treated with IUT | 8 | January 2001–December 2001 | Pakistan |
|  |  |  | Hydropic fetuses, D-mediated HDFN treated with IUT | 4 |  |  |
|  |  |  | Non-hydropic fetuses, D-mediated HDFN treated with IUT | 4 |  |  |
|  |  |  | Neonates, D-mediated HDFN treated with IUT | 5 |  |  |
| Navarro 2009^50^ | Case series | 3 | Neonate, D-mediated HDFN (case #3) | 1 | NR | USA |
| Nwogu 2018^51^ | Case series | 5 | Fetuses, D-mediated HDFN  Fetus, D- and K-mediated HDFN  Fetus, K-mediated HDFN | 3  1  1 | November 2011–December 2015 | USA |
| Palfi 2006^52^ | Case report | 1 | Pregnancy, D-mediated HDFN | 1 | NA | Sweden |
|  |  |  | Neonate, D-mediated HDFN | 1 |  |  |
| Phung 2018^53^ | Retrospective cohort | 106 | Fetuses, isolated D-mediated HDFN treated with IUT | 29 | 1999–2015 | France |
|  |  |  | Neonates, isolated D-mediated HDFN treated with IUT | 27 |  |  |
|  |  |  |  |  |  |  |
| Raguz 2020^2^ | Retrospective cohort | 29,663 pregnancies, 545 pos antibodies, 175 anti-D, 21 Kell 310 newborns with HDFN, 59 anti-D newborns, 5 Kell newborns | Infants, D-mediated HDFN | 59 | 2000–2019 | Bosnia and Herzegovina |
|  |  |  | Infants, K-mediated HDFN | 5 |  |  |
| Rahimi-Levene 2020^54^ | Retrospective cohort | 90,948 | Pregnant women, D-mediated HDFN | 63 | January 1, 2011–December 31, 2011 | Israel |
|  |  |  | Pregnant women, K-mediated HDFN | 114 |  |  |
| Rahimi-Sharbaf 2007^55^ | Case report | 1 | Pregnancy, D-mediated HDFN | 1 | NA | Iran |
| Rath 2011^56^ | Retrospective cohort | 191 | (Near)-term neonates, D-mediated HDFN  (Near)-term neonates, K-mediated HDFN | 157  34 | January 2000–December  2008 | The Netherlands |
|  |  |  | Neonate, D-mediated HDFN | 1 |  |  |
| Rath 2012^57^ | Retrospective cohort | 362 | Infants, D-mediated HDFN | 268 | January 2000–September 2010 | The Netherlands |
|  |  |  | Infants, K-mediated HDFN | 51 |  |  |
| Rath 2013^58^ | Observational cohort | 35 | Neonate, D-mediated HDFN | 1 | November 2010–March 2012 | The Netherlands |
|  |  |  | Neonates, HDFN and cholestasis | 5 |  |  |
| Rath 2013^59^ | Retrospective cohort | 125 | Neonates, D-mediated HDFN | 103 | January 2000–October 2011 | The Netherlands |
| Ree 2019^60^ | Observational cohort | 298 | Infants, D-mediated HDFN | 224 | January 2006–January 2018 | The Netherlands |
|  |  |  | Infants, D-mediated HDFN treated with IUT | 148 |  |  |
|  |  |  | Infants, D-mediated HDFN not treated with IUT | 76 |  |  |
|  |  |  | Infants, D-mediated HDFN treated with IUT and RBC transfusion | 134 |  |  |
|  |  |  | Infants, D-mediated HDFN treated with IUT and not with RBC transfusion | 14 |  |  |
|  |  |  | Infants, D-mediated HDFN not treated with IUT and with RBC transfusion | 53 |  |  |
|  |  |  | Infants, D-mediated HDFN not treated with IUT or RBC transfusion | 23 |  |  |
|  |  |  | Infants, K-mediated HDFN | 39 |  |  |
| Ree 2020^61^ | Observational cohort | 235 | Fetuses, D- or K-mediated HDFN | 235 | January 2005–December 2018 | The Netherlands |
|  |  |  | Fetuses, D-mediated HDFN | 189 |  |  |
|  |  |  | Fetuses, K-mediated HDFN | 46 |  |  |
|  |  |  | Neonates, D-mediated HDFN | 189 |  |  |
|  |  |  | Neonates, K-mediated HDFN | 46 |  |  |
| Ree 2020^62^ | Retrospective cohort | 317 | Infant, D-mediated HDFN and NEC (case #1) | 1 | January 2000–December 2016 | The Netherlands |
|  |  |  | Infant, D-mediated HDFN and NEC (case #2) | 1 |  |  |
|  |  |  | Infant, K-mediated HDFN and NEC (case #4) | 1 |  |  |
| Ruma 2007^63^ | Multicenter case series | 9 | Pregnancies, D- or K-mediated HDFN | 9 | 1996–2005 | USA |
|  |  |  | Pregnancies, D-mediated HDFN | 5 |  |  |
|  |  |  | Pregnancies, K-mediated HDFN | 4 |  |  |
|  |  |  | Infants, D- or K-mediated HDFN | 9 |  |  |
| Sainio 2015^64^ | Retrospective cohort | 104 | Pregnancies, K-mediated HDFN treated with IUT | 8 | 2003–2012 | Finland |
|  |  |  | Pregnancies, D-mediated HDFN treated with IUT | 86 |  |  |
|  |  | 339 | IUTs | 339 |  |  |
|  |  |  | Intrauterine deaths associated with IUTs | 4 |  |  |
| Santos 2013^65^ | RCT | 92 | Infants, D-mediated HDFN receiving IVIG | 46 | April 2006–June 2009 | Brazil |
|  |  |  | Infants, D-mediated HDFN receiving placebo | 46 |  |  |
|  |  |  | Infants, D-mediated HDFN receiving IVIG, no IUT | 42 |  |  |
|  |  |  | Infants, D-mediated HDFN receiving placebo, no IUT | 40 |  |  |
| Şavkli 2020^66^ | Retrospective cohort | 110 | IUTs  IUTs in pregnancies where fetuses were hydropic at first IUT, D-mediated HDFN | 51 | January 2015–July 2018 | Turkey |
|  |  |  | IUTs in pregnancies where fetuses were not hydropic at first IUT, D-mediated HDFN | 59 |  |  |
|  |  | 42 | Pregnancies treated with IUT, D-mediated HDFN | 42 |  |  |
| Sikkel 2005^67^ | Prospective cohort | 49 transfusions, 23 fetuses | IUTs in D-mediated HDFN group | 41 | March 2001–May 2002 | The Netherlands |
|  |  |  | IUTs in K-mediated HDFN group | 3 |  |  |
| Simonazzi 2016^68^ | Case series | 4 | Fetus, D-mediated HDFN (case #1) | 1 | NR | Italy |
|  |  |  | Fetus, D-mediated HDFN (case #2) | 1 |  |  |
|  |  |  | Fetus, D-mediated HDFN (case #3) | 1 |  |  |
| Somerset 2006^69^ | Retrospective cohort | 66 | Anemic fetuses, D-mediated HDFN | 57 | March 1997–March 2004 | USA |
|  |  |  | Anemic fetuses, K-mediated HDFN | 7 |  |  |
| Takci 2013^70^ | Retrospective cohort | 30 | Hydropic infants, D-mediated HDFN | 30 | January 2001–June 2012 | Turkey |
|  |  |  | Hydropic infants, D-mediated HDFN, non-cholestasis group | 12 |  |  |
|  |  |  | Hydropic infants, D-mediated HDFN, cholestasis group | 18 |  |  |
| Tara 2019^71^ | Case report | 1 | Fetus, D-mediated HDFN | 1 | NR | Iran |
| Temel 2019^72^ | Prospective cohort | 17 | Pregnant women, D-mediated HDFN | 17 | January 2018–June 2019 | Turkey |
|  |  |  | Neonates, D-mediated HDFN | 17 |  |  |
| Tiblad 2011^73^ | Retrospective cohort | 84 | Fetuses, D-mediated HDFN | 67 | June 1990–June 2010 | Sweden |
|  |  |  | Fetuses, K-mediated HDFN | 9 |  |  |
|  |  |  | Fetus, D-mediated HDFN (case #511) | 1 |  |  |
|  |  |  | Fetus, D-mediated HDFN (case #512) | 1 |  |  |
|  |  |  | Fetus, D-mediated HDFN (case #514) | 1 |  |  |
|  |  |  | Fetus, D-mediated HDFN (case #518; 1991 pregnancy) | 1 |  |  |
|  |  |  | Fetus, D-mediated HDFN (case #518; 1993 pregnancy) | 1 |  |  |
|  |  |  | Fetus, D-mediated HDFN (case #249) | 1 |  |  |
|  |  |  | Fetus, D-mediated HDFN (case #462) | 1 |  |  |
|  |  |  | Fetus, D-mediated HDFN (case #459) | 1 |  |  |
|  |  |  | Fetus, D-mediated HDFN (case #343) | 1 |  |  |
| Urutherakumar 2020^74^ | Retrospective cohort | 39 | Transfused fetus (case #4) | 1 | January 1, 2005–July 30, 2017 | Australia |
| Van Den Akker 2008^75^ | Retrospective cohort | 42 | Fetuses, K-mediated HDFN | 42 | January 1988–December 2005 | The Netherlands |
|  |  |  | Severely hydropic fetuses, K-mediated HDFN | 15 |  |  |
| Walsh 2013^76^ | Retrospective cohort | 102 | Fetuses, D-mediated HDFN | 26 | January 1, 1996–December 31, 2011 | Ireland |
|  |  |  | Fetuses, K-mediated HDFN | 11 |  |  |
|  |  |  |  |  |  |  |
| Xu 2013^77^ | Case report | 1 | Neonate | 1 | NA | PRC |
| Zwiers 2017^78^ | Retrospective cohort | 589 | Neonate | 1 | January 1988–January 2015 | The Netherlands |
| Zwiers 2018^79^ | Retrospective cohort | 645 | Fetuses, D-mediated HDFN, | 524 | January 1, 1987–December 31, 2016 | The Netherlands |
|  |  |  | Fetuses, K-mediated HDFN | 83 |  |  |
|  |  |  | Fetuses, D-mediated HDFN, 1999 onward cohort | 334 |  |  |
|  |  |  | Fetuses, K-mediated HDFN, 1999 onward cohort | 65 |  |  |
| Zwiers 2018^80^ | Retrospective multicenter cohort | 52 | Women (D- or K-mediated HDFN) managed with IVIG, unadjusted data | 24 | January 2010–June 2016 | The Netherlands |
|  |  |  | Women (D- or K-mediated HDFN) managed without IVIG, unadjusted data | 28 |  |  |
|  |  |  | Women (D- or K-mediated HDFN) managed with IVIG, weighted data^a^ | 24 |  |  |
|  |  |  | Women (D- or K-mediated HDFN) managed without IVIG, weighted data^a^ | 25 |  |  |

D, Rh(D); HDFN, hemolytic disease of the fetus and newborn; IUT, intrauterine transfusion; IVIG, intravenous immunoglobulin; K, Kell; NA, not applicable; NEC, necrotizing enterocolitis; NR, not reported; PRC, People’s Republic of China; RCT, randomized controlled trial; RBC, red blood cell; Rh, Rhesus; USA, United States of America.

For D- or K-mediated HDFN, data were captured only for cases with immunization types of D only, K only, or both D and K only.

^a^Data were corrected for the probability that women would be selected for IVIG treatment by their caregivers in the index pregnancy. Propensity scores were calculated using gestational age at onset of anemia in the previous pregnancy, interval between pregnancies, number of previous births, type of alloimmunization, maternal body mass index, and number of IUTs in the previous pregnancy.

**Table S2. Research Questions Relevant to Mothers and Fetuses**

| Treatment landscape | What is the current treatment landscape of HDFN in mothers and fetuses? |
| --- | --- |
|  | What are the commonly used treatments? |
| Clinical evidence of HDFN treatments | What are the maternal and fetal clinical outcomes? |
|  | What is the efficacy of intravenous immunoglobulin in women with HDFN? |
|  | What are the complications of treatment (eg, adverse events)? |
|  | What is the prevalence of HDFN, and what is the prevalence of early-onset HDFN^a^? |
| Evidence gaps and recommendations | What are the evidence gaps in the HDFN literature? |
|  | What recommendations can be proposed for future research? |

HDFN, hemolytic disease of the fetus and newborn.

^a^HDFN requiring intervention before 24 weeks of pregnancy.

**Table S3. Inclusion and Exclusion Criteria^a^**

|  | Inclusion Criteria | Exclusion Criteria |
| --- | --- | --- |
| Population | - Women and/or their fetus/infant/child experiencing or having experienced HDFN caused by Rh incompatibility - Antigen status: Rh(D) or Kell antigen | - Women and fetus/infant/child who did not experience HDFN with ABO incompatibility - Antigen status: c, e, E, Duffy (Fy), Kidd (Jk), MNS (S), or Gerbich antigen |
| Intervention | - Any or none | - Not applicable |
| Comparator | - Any or none | - Not applicable |
| Outcomes | - Treatment patterns - Clinical outcomes - Treatment efficacy | - Not applicable |
| Study design | - Observational studies (retrospective or prospective) including cohort, case-control, or cross-sectional studies - Trials (randomized or nonrandomized) - Modelling studies - Systematic reviews of cohort studies (for identification of primary studies only) - Case reports and case series | - Notes, editorials, or commentaries - Nonsystematic reviews |
| Other | - Journal articles - Human subjects - English language - Studies published between January 1, 2005, and March 10, 2021 | - Indexed conference abstracts - Publication types not of interest - Animal or preclinical studies - Non-English language - Studies published before January 1, 2005 |

HDFN, hemolytic disease of the fetus and newborn; Rh, Rhesus.

^a^Population, intervention, comparison, outcomes, and study (PICOS) design was used as a framework to formulate eligibility criteria.^61^

**Table S4a.** Methodologic Quality of Selected Case Reports

| **JBI Critical Appraisal Checklist for Case Reports** | | | | | | | | | |
| --- | --- | --- | --- | --- | --- | --- | --- | --- | --- |
|  | **Q1** | **Q2** | **Q3** | **Q4** | **Q5** | **Q6** | **Q7** | **Q8** | **Total Score** |
| **Akdağ et al** | **Yes** | **Yes** | **Yes** | **Yes** | **Yes** | **No** | **Yes** | **Yes** | **7/8** |
| **Bek et al** | **Yes** | **No** | **Yes** | **Yes** | **Yes** | **Yes** | **Yes** | **Yes** | **7/8** |
| **Brumbaugh et al** | **Yes** | **Yes** | **Yes** | **Yes** | **Yes** | **Yes** | **Yes** | **Yes** | **8/8** |
| **Colpo et al** | **Yes** | **Yes** | **Yes** | **Yes** | **Yes** | **Yes** | **Yes** | **Yes** | **8/8** |
| **Fernández Alba et al** | **Yes** | **Yes** | **Yes** | **Yes** | **Yes** | **No** | **Unc** | **Yes** | **6/8** |
| **Haider et al** | **Yes** | **No** | **Yes** | **Yes** | **Yes** | **Yes** | **Unc** | **Yes** | **6/8** |
| **Hassan et al** | **No** | **Yes** | **No** | **Yes** | **No** | **Yes** | **Yes** | **Yes** | **5/8** |
| **Lakhwani et al** | **Yes** | **Yes** | **Yes** | **Yes** | **Yes** | **Yes** | **Yes** | **Yes** | **8/8** |
| **Levy-Zauberman et al** | **Yes** | **Yes** | **Yes** | **Yes** | **Yes** | **Yes** | **Yes** | **Yes** | **8/8** |
| **Manoura et al** | **No** | **Unc** | **No** | **Yes** | **Yes** | **Unc** | **Unc** | **Yes** | **3/8** |
| **Palfi et al** | **Yes** | **Yes** | **Yes** | **Yes** | **Yes** | **Yes** | **Yes** | **Yes** | **8/8** |
| **Rahimi-Sharbaf et al** | **Yes** | **Yes** | **No** | **Yes** | **No** | **No** | **Unc** | **Yes** | **4/8** |
| **Tara et al** | **Yes** | **Yes** | **Yes** | **Yes** | **Yes** | **Yes** | **Yes** | **Yes** | **8/8** |
| **Xu et al** | **Yes** | **Yes** | **Yes** | **Yes** | **Yes** | **Yes** | **Yes** | **Yes** | **8/8** |
| JBI, Joanna Briggs Institute; Unc, unclear.  Q1: Were patient’s demographic characteristics clearly described?  Q2: Was the patient’s history clearly described and presented as a timeline?  Q3: Was the current clinical condition of the patient on presentation clearly described?  Q4: Were diagnostic tests or assessment methods and the results clearly described?  Q5: Was the intervention(s) or treatment procedure(s) clearly described?  Q6: Was the post-intervention clinical condition clearly described?  Q7: Were adverse events (harms) or unanticipated events identified and described?  Q8: Does the case report provide takeaway lessons? | | | | | | | | | |

**Table S4b.** Methodologic Quality of Selected Case Series

| **JBI Critical Appraisal Checklist for Case Series** | | | | | | | | | | | |
| --- | --- | --- | --- | --- | --- | --- | --- | --- | --- | --- | --- |
|  | **Q1** | **Q2** | **Q3** | **Q4** | **Q5** | **Q6** | **Q7** | **Q8** | **Q9** | **Q10** | **Total Score** |
| **Fox et al** | **Yes** | **Yes** | **Yes** | **Yes** | **Yes** | **Yes** | **Yes** | **Yes** | **Yes** | **NA** | **9/9** |
| **Kriplani et al** | **No** | **Yes** | **Yes** | **Yes** | **Unc** | **Yes** | **Yes** | **Unc** | **No** | **NA** | **5/9** |
| **Mayer et al** | **No** | **Yes** | **Yes** | **Unc** | **Unc** | **Yes** | **Yes** | **Unc** | **No** | **NA** | **4/9** |
| **Navarro et al** | **No** | **Yes** | **Yes** | **Unc** | **Unc** | **Yes** | **Yes** | **Yes** | **No** | **NA** | **5/9** |
| **Nwogu et al** | **Yes** | **Yes** | **Yes** | **Yes** | **Yes** | **Yes** | **Yes** | **No** | **Yes** | **NA** | **8/9** |
| **Ruma et al** | **Yes** | **Yes** | **Yes** | **Yes** | **Yes** | **Yes** | **Yes** | **Yes** | **Yes** | **Yes** | **10/10** |
| **Simonazzi et al** | **No** | **Yes** | **Yes** | **Unc** | **Unc** | **Yes** | **Yes** | **Yes** | **No** | **NA** | **5/9** |
| JBI, Joanna Briggs Institute; Unc, unclear.  NA = Not applicable  Q1: Were there clear criteria for inclusion in the case series?  Q2: Was the condition measured in a standard, reliable way for all participants included in the case series?  Q3: Were valid methods used for identification of the condition for all participants included in the case series?  Q4: Did the case series have consecutive inclusion of participants?  Q5: Did the case series have complete inclusion of participants?  Q6: Was there clear reporting of the demographics of the participants in the study?  Q7: Was there clear reporting of clinical information of the participants?  Q8: Were the outcomes or follow up results of cases clearly reported?  Q9: Was there clear reporting of the presenting site(s)/clinic(s) demographic information?  Q10: Was statistical analysis appropriate? | | | | | | | | | | | |

**Table S4c.** Methodologic Quality of Selected Retrospective Cohort Studies

| **Newcastle-Ottawa Scale (Retrospective Cohort Studies)** | | | | | | | | | | |
| --- | --- | --- | --- | --- | --- | --- | --- | --- | --- | --- |
|  | **Selection** | | | | **Comparability** | **Outcome** | | |  |  |
|  | **I1** | **I2** | **I3** | **I4** | **I5** | **I6** | **I7** | **I8** | **Total Score** | **Quality** |
| **Al-Dughaishi 2016** | ***** | ***** | ***** | ***** |  | ***** |  |  | **5** | **Poor** |
| **Åžavkli 2020** | ***** | ***** | ***** | ***** | ***** | ***** | ***** | ***** | **8** | **Good** |
| **Canlorbe 2011** | ***** | ***** | ***** | ***** |  | ***** | ***** | ***** | **7** | **Fair** |
| **Chatziantoniou 2017** | ***** | ***** | ***** | ***** | ***** | ***** | ***** | ***** | **8** | **Good** |
| **Gottvall 2008** | ***** | ***** | ***** | ***** | ***** | ***** | ***** | ***** | **8** | **Good** |
| **Gudlaugsson 2020** | ***** | ***** | ***** | ***** | ***** | ***** | ***** | ***** | **8** | **Good** |
| **Karagol 2012** | ***** | ***** | ***** | ***** | ***** | ***** | ***** | ***** | **8** | **Good** |
| **Lieberman 2020** | ***** | ***** | ***** | ***** | ***** | ***** | ***** | ***** | **8** | **Good** |
| **Matijevic 2005** | ***** |  | ***** | ***** |  | ***** | ***** | ***** | **6** | **Fair** |
| **Meraj 2015** | ***** |  | ***** | ***** |  | ***** | ***** | ***** | **6** | **Fair** |
| **Phung 2018** | ***** | ***** | ***** | ***** |  | ***** | ***** | ***** | **7** | **Fair** |
| **Rahimi-Levene 2020** | ***** | ***** | ***** | ***** |  | ***** | ***** | ***** | **7** | **Fair** |
| **Rath 2012** | ***** | ***** | ***** | ***** | ***** | ***** | ***** | ***** | **8** | **Good** |
| **Rath 2013a** | ***** | ***** | ***** | ***** | ***** | ***** | ***** | ***** | **8** | **Good** |
| **Rath 2013b** | ***** | ***** | ***** | ***** | ***** | ***** | ***** | ***** | **8** | **Good** |
| **Ree 2019** | ***** | ***** | ***** | ***** | ***** | ***** | ***** | ***** | **8** | **Good** |
| **Ree 2020a** | ***** | ***** | ***** | ***** | ***** | ***** | ***** | ***** | **8** | **Good** |
| **Ree 2020b** | ***** | ***** | ***** | ***** | ***** | ***** | ***** | ***** | **8** | **Good** |
| **Sainio 2015** | ***** | ***** | ***** | ***** | ***** | ***** | ***** | ***** | **8** | **Good** |
| **Somerset 2006** | ***** | ***** | ***** | ***** | ***** | ***** | ***** | ***** | **8** | **Good** |
| **Takci 2013** | ***** | ***** | ***** | ***** |  | ***** | ***** | ***** | **7** | **Fair** |
| **Tiblad 2011** | ***** | ***** | ***** | ***** | ***** | ***** | ***** | ***** | **8** | **Good** |
| **Urutherakumar 2020** | ***** | ***** | ***** | ***** |  | ***** | ***** | ***** | **7** | **Fair** |
| **Van Den Akker 2008a** | ***** | ***** | ***** | ***** | ***** | ***** | ***** | ***** | **8** | **Good** |
| **Walsh 2013** | ***** | ***** | ***** | ***** | ***** | ***** | ***** | ***** | **8** | **Good** |
| **Zwiers 2017** | ***** | ***** | ***** | ***** | ***** | ***** | ***** | ***** | **8** | **Good** |
| **Zwiers 2018a** | ***** | ***** | ***** | ***** | ***** | ***** | ***** | ***** | **8** | **Good** |
| **Rath 2011** | ***** | ***** | ***** | ***** | ***** | ***** | ***** | ***** | **8** | **Good** |
| **Raguz 2020** | ***** | ***** | ***** | ***** |  | ***** | ***** | ***** | **7** | **Fair** |
| **Zwiers 2018b** | ***** | ***** | ***** | ***** | ***** | ***** | ***** | ***** | **8** | **Good** |
| I1: Representativeness of the exposed cohort  I2: Selection of the non-exposed cohort  I3: Ascertainment of exposure  I4: Demonstration that outcome of interest was not present at start of study  I5: Comparability of cohorts on the basis of the design or analysis controlled for confounders  I6: Assessment of outcome  I7: Was follow-up long enough for outcomes to occur  I8: Adequacy of follow-up of cohorts | | | | | | | | | | |

**Table S4d.** Methodologic Quality of Selected Prospective Cohort Studies

| **Newcastle-Ottawa Scale (Prospective Cohort Studies)** | | | | | | | | | | |
| --- | --- | --- | --- | --- | --- | --- | --- | --- | --- | --- |
|  | **Selection** | | | | **Comparability** | **Outcome** | | |  |  |
|  | **I1** | **I2** | **I3** | **I4** | **I5** | **I6** | **I7** | **I8** | **Total Score** | **Quality** |
| **Bi 2019** | ***** |  | ***** | ***** | ***** | ***** | ***** | ***** | **7** | **Good** |
| **Craparo 2005** | ***** |  | ***** | ***** | ***** | ***** | ***** | ***** | **7** | **Good** |
| **de Assunção 2016** | ***** | ***** | ***** | ***** | ***** | ***** | ***** | ***** | **8** | **Good** |
| **Dubey 2016** | ***** | ***** | ***** | ***** | ***** | ***** | ***** | ***** | **8** | **Good** |
| **Sikkel 2005** | ***** | ***** | ***** | ***** | ***** | ***** | ***** | ***** | **8** | **Good** |
| **Temel 2019** | ***** | ***** | ***** | ***** | ***** | ***** | ***** | ***** | **8** | **Good** |
| **Harper 2006** | ***** | ***** | ***** | ***** | ***** | ***** | ***** | ***** | **8** | **Good** |
| **Koelewijn 2008** | ***** | ***** | ***** | ***** | ***** | ***** | ***** | ***** | **8** | **Good** |
| I1: Representativeness of the exposed cohort  I2: Selection of the non-exposed cohort  I3: Ascertainment of exposure  I4: Demonstration that outcome of interest was not present at start of study  I5: Comparability of cohorts on the basis of the design or analysis controlled for confounders  I6: Assessment of outcome  I7: Was follow-up long enough for outcomes to occur  I8: Adequacy of follow-up of cohorts | | | | | | | | | | |

**Table S4e.** Methodologic Quality of Bennardello et al(25)

| **Item** | **Bennardello et al** |
| --- | --- |
| **Design** |  |
| ***Is the target population defined?*** | **Yes, all Italian Transfusion Structures** |
| **IRB approval and informed consent process** |  |
| ***IRB approval*** | **No report** |
| ***Informed consent*** | **No report** |
| ***Data protection*** | **No report** |
| **Development and pre-testing** |  |
| ***Development and testing*** | **The SIMTI set up a Working Group to design a multiple choice questionnaire** |
| **Recruitment process and description of the sample having access to the questionnaire** |  |
| ***Open survey versus closed survey*** | **Closed survey** |
| ***Contact mode*** | **Website, fax, or email** |
| ***Advertising the survey*** | **Internal communication within the organization** |
| **Survey administration** |  |
| ***Web/e-mail*** | **Online entry was possible** |
| ***Context*** | **SIMTI website, specifically for Italian Transfusion Structures** |
| ***Mandatory/voluntary*** | **Voluntary** |
| ***Incentives*** | **No incentives reported** |
| ***Time/date*** | **2011** |
| ***Randomization of items or questionnaires*** | **NA** |
| ***Adaptive questioning*** | **No report** |
| ***Number of items*** | **37 questions** |
| ***Number of screens (pages)*** | **5 sections** |
| ***Completeness check*** | **The mean percentage of questions answered was 76%** |
| ***Review step*** | **No report** |
| **Response rates** |  |
| ***Unique site visitor*** | **The 280 Italian Transfusion Structures were invited via the SIMTI website** |
| ***View rate (ratio of unique survey visitors/unique site visitors)*** | **NA** |
| ***Participation rate (ratio of unique visitors who agreed to participate/unique first survey page visitors)*** | **176/280 overall (participation rate differed be region)** |
| ***Completion rate (ratio of users who finished the survey/users who agreed to participate)*** | **164/280** |
| **Preventing multiple entries from the same individual** |  |
| ***Cookies used*** | **No report** |
| ***IP check*** | **No report** |
| ***Log file analysis*** | **No report** |
| ***Registration*** | **Yes, per Italian Transfusion Structure** |
| **Analysis** |  |
| ***Handling of incomplete questionnaires*** | **Incomplete questionnaires were included for analysis** |
| ***Questionnaires submitted with an atypical timestamp*** | **No report** |
| ***Statistical correction*** | **NA** |
| IRB, institutional review board; NA, not applicable; SIMTI, The Italian Society of Transfusion Medicine and Immunohaematology. | |

**Table S5.** Antenatal Treatments Reported in Studies With Representative Data

| Citation | Population | D Only and/or K Only^a^ | | Treatment (%)^b^ |
| --- | --- | --- | --- | --- |
| **IUT Only** |  |  | |  |
| Santos 2013^45^ | 4/46 infants treated with IVIG^b,c^  6/46 infants given placebo^c^ | D  D | --  -- | 8.7*  13.0* |
| Dubey 2016^12^ | 17/29 | D | -- | 58.6 |
| Chatziantoniou 2017^8^ | 2/32 | D | -- | 6.3* |
| Gottvall 2008^15^ | 0/9 | -- | K | 0 |
| Gudlaugsson 2020^16^ | 5/112 | D | -- | 4.5* |
| Rath 2011^36^ | 104/157  28/34 | D  -- | --  K | 66  82 |
| Rath 2013^39^ | 61/103 | D | -- | 59 |
| Ree 2019^40^ | 148/224  35/39 | D  -- | --  K | 66  90 |
| Bennardello 2013^4^ | 8/111  1/8 | D  -- | --  K | 7.2  12.5 |
| **IVIG Only** |  |  |  |  |
| Mayer 2018^27^ | 1/1  1/1 | D  -- | --  K | 100  100 |
| **IUT+IVIG** |  |  |  |  |
| Ree 2020^42^ | 6/189 treated with IUT+IVIG^d^ | D | -- | 3.2* |
|  | 2/46 treated with IUT+IVIG^c^ | -- | K | 4.3* |
| **Other** |  |  |  |  |
| Tara 2019^51^ | 1/1 | D | -- | TPE, 100 |
| Gottvall 2008^15^ | 10/71 | D | -- | Maternal plasma exchange  and/or high-dose IVIG, 14.1* |
| Nwogu 2018^30^ | 5/5 | D and/or K | | TPE, IVIG, and IUT, 100 |
| Palfi 2006^31^ | 1/1 | D | -- | TPE, IVIG, and IUT,100 |
| Colpo 2017^9^ | 1/1 | D | -- | TPE, immunoadsorption, IVIG, and IUT, 100 |
| Lakhwani 2011^22^ | 1/1 | -- | K | Plasmapheresis and IUT, 100 |
| Fernandez Alba 2014^13^ | 1/1 | D | -- | Plasmapheresis and IVIG, 100 |
| Zwiers 2018^60^ | 8/24 | D or K | | Plasmapheresis, IVIG, and IUT, 33.3* |
| Ruma 2007^43^ | 9/9 | D or K | | Plasmapheresis, IVIG, and IUT, 100 |
| Bek 2019^3^ | 1/1 | D | -- | Plasmapheresis, IVIG, and IUT, 100 |

D, Rh(D); HDFN, hemolytic disease of the fetus and newborn; IUT, intrauterine transfusion; IVIG, intravenous immunoglobulin; K, Kell; Rh, Rhesus; TPE, therapeutic plasma exchange.

^a^Data were captured only for cases with immunization types of D only, K only, or both D and K only.

^b^Values are as provided in reports, unless otherwise noted (*values that were calculated).

^c^Studied to evaluate the need for exchange transfusion among neonates randomly allocated to treatment with or without IVIG (both groups had intensive phototherapy).

^d^Studied for suppression of fetal erythropoiesis.

**Table S6.** Hydrops Fetalis, Severe Fetal Anemia, and Adverse Events/Complications Reported in Studies With Representative Data

| Citation | Patient Group | N | D Only and/or  K Only^a^ | | Events (n/N [%]^b^) | |
| --- | --- | --- | --- | --- | --- | --- |
| **Hydrops Fetalis** |  |  |  |  |  | |
| Craparo 2005^10^ | Pregnancies treated with IUT | 31 | D | -- | 7/31 (23) | |
| de Assuncão 2016^11^ | Pregnancies treated with IUT | 13 | D | -- | 3/13 (23.1) | |
| Sikkel 2005^47^ | IUTs | 41 | D | -- | 4/41 (9.8) | |
|  | IUTs | 3 | -- | K | 2/3 (66.7) | |
| Al-Dughaishi 2016^2^ | Pregnant women | 8 | D and/or K | | 0/8 (0) | |
| Canlorbe 2011^7^ | IUTs performed <20 weeks’ gestation | 25 | D or K | | 11/25 (44)* | |
| Karagol 2012^20^ | Neonates | 5 | -- | K | 0/5 (0) | |
| Matijevic 2005^26^ | Pregnancies | 15  1 | D  -- | --  K | 3/15 (18.8)*  0/1 (0) | |
| Meraj 2015^28^ | Fetuses treated with IUT | 8 | D | -- | 4/8 (50) | |
|  | Fetuses treated with IUT, hydropic | 4 | D | -- | 4/4 (100) | |
|  | Fetuses treated with IUT, non-hydropic | 4 | D | -- | 0/4 (0) | |
| Phung 2018^32^ | Fetuses treated with IUT for isolated D-mediated HDFN | 29 | D | -- | 4/29 (13.8) | |
| Rath 2013^39^ | Neonates | 103 | D | -- | 2/103 (1.9) | |
| Somerset 2006^49^ | Pregnancies complicated by fetal anemia requiring IUT | 57 | D | -- | 15/57 (26.3)* | |
|  |  | 7 | -- | K | 2/7 (28.6)* | |
| Sainio 2015^44^ | Pregnancies | 104 | D or K | | 12/104 (11.5) | |
|  |  | 8 | -- | K | 3/8 (37.5) | |
|  | Intrauterine deaths associated with IUTs | 3 | D | -- | 1/3 (33.3)* | |
|  |  | 1 | -- | K | 1/1 (100)* | |
| Şavkli 2020^46^ | Pregnancies treated with IUT | 42 | D | -- | 14/42 (33.3) | |
|  | IUTs in pregnancies where fetuses were hydropic at first IUT | 51 | D | -- | 51/51 (100) | |
|  | IUTs in pregnancies where fetuses were not hydropic at first IUT | 59 | D | -- | 0/59 (0) | |
| van den Akker 2008^55^ | Fetuses | 42 | -- | K | 15/42 (36) (severe) 9/42 (21) (mild)* | |
|  | Severely hydropic fetuses | 15 | -- | K | 15/15 (100) (severe) | |
| Walsh 2013^56^ | Fetuses | 26 | D | -- | 0/26 (0) | |
|  |  | 11 | -- | K | 2/11 (17) | |
| Zwiers 2018^59^ | Fetuses, 1999 onward cohort | 334 | D | -- | 40/338 (12) | |
|  | Fetuses, 1999 onward cohort | 65 | -- | K | 21/65 (32) | |
| Zwiers 2018^60^ | Unadjusted data, IVIG group | 24 | D or K | | 1/24 (4) | |
|  | Unadjusted data, non-IVIG group | 28 | D or K | | 7/28 (24) | |
|  | Weighted data,^c^ IVIG group | 24 | D or K | | 1/24 (4) | |
|  | Weighted data,^c^ non-IVIG group | 25 | D or K | | 12/25 (46) | |
| Kriplani 2007^21^ | Pregnancies | 4 | D | -- | 2/4 (50)* | |
| Nwogu 2018^30^ | Fetuses, D-mediated HDFN, antenatal treatment with IVIG and IUT | 3 | D | -- | 0/3 (0) | |
|  | Fetuses, D- and K-mediated HDFN | 1 | D | K | 0/1 (0) | |
|  | Fetuses, K-mediated HDFN | 1 | -- | K | 0/1 (0) | |
| **Severe Fetal Anemia^d^** |  |  |  |  |  | |
| Canlorbe 2011^7^ | Pregnancies with IUTs performed/attempted ≤20 weeks’ gestation | 18 | D or K | | 18/18 (100) | |
| **Adverse Events/Complications** | |  |  | |  |  |
| Dubey 2016^12^ | Pregnancies | 29 | D | -- | Adverse serological outcomes, 17/29 (58.8) | |
| Temel Yüksel 2019^52^ | Fetuses treated with IUT | 17 | D | -- | non-reassuring fetal heart rate tracing, 3/17 (17.6) | |
| Canlorbe 2011^7^ | IUTs performed <20 weeks’ gestation | 25 | D or K | | Failed intravascular transfusion, 2/25 (8)*; bradycardia, 1/25 (4)* | |
|  |  |  |  |  |  | |
| Matijevic 2005^26^ | Pregnancies | 15 | D | -- | Fetal death, 2/15 (13.3)* | |
|  |  | 1 | -- | K | Fetal death, 0/1 (0) | |
| Sainio 2015^44^ | Pregnancies | 104  86 | D or K | | Fetal death, 4/104 (3.8)  Fetal death, 3/86 (3.5) | |
|  |  |  | D | -- |  | |
|  |  | 8 | -- | K | Fetal death, 1/8 (1.3) | |
|  |  | 104 | D or K | | Severe fetal bradycardia requiring immediate cesarean section, 11/104 (10.6) | |
|  |  | 104 | D or K | | Infection, PROM, or spontaneous preterm  delivery <32 weeks of gestation, 9/104 (8.7) | |
|  | IUTs | 339 | D or K | | Fetal death, 4/339 (1.2) | |
|  |  | 339 | D or K | | Severe fetal bradycardia requiring immediate | |
|  |  |  |  |  | cesarean section, 11/339 (3.2) | |
|  |  | 339 | D or K | | Infection, PROM, or spontaneous preterm delivery <32 weeks of gestation, 9/339 (2.7)* | |
|  | Intrauterine deaths associated with IUTs | 3 | D | -- | PROM, 1/3 (33.3)*; infection, 1/3 (33.3)* | |
|  |  | 1 | -- | K | 0/1 (0) | |
| Şavkli 2020^46^ | IUTs | 110 | D | -- | 13-110 (11.8) (PROM, 0/110; preterm labor; 2/110 (1.8)*;  fetal distress, 5/110 (4.5)*; fetal death, 6/110 (5.5)* | |
| Somerset 2006^49^ | Anemic fetuses | 57 | D | -- | Bradycardia, 7/57 (12.3)*; spontaneous rupture of membranes, 3/57 (5.3)*; chorioamnionitis, 1/57 (1.8)*; immediate delivery 3/57 (5.3)* | |
|  | Anemic fetuses | 7 | -- | K | Bradycardia, 1/7 (14.3)*; spontaneous rupture of membranes, 0/7 (0); chorioamnionitis, 1/7 (14.3)*; immediate delivery, 0/7 (0) | |
| Zwiers 2018^60^ | Unadjusted data, women managed without IVIG | 28 | D or K | | PROM/premature delivery, 1/28 (3.7) | |
| Ruma 2007^43^ | Pregnancies | 9 | D or K | | 4/9 (44) (IVIG associated: headache, 2/9 [22.2]*; chest congestion, 1/9 [11.1]*; mild flushing and pruritis, 1/9 [11.1]*; plasmapheresis associated: 0/9 [0]) | |

D, Rh(D); HDFN, hemolytic disease of the fetus and newborn; IUT, intrauterine transfusion; IVIG, intravenous immunoglobulin; K, Kell; NICU, neonatal intensive care unit; PROM, premature rupture of membranes; Rh, Rhesus; SD, standard deviation.

^a^Data were captured only for cases with immunization types of D only, K only, or both D and K only.

^b^Values are as provided in reports, unless otherwise noted (*for values that were calculated).

^c^Data were corrected for the probability that women would be selected for IVIG treatment by their caregivers in the index pregnancy. Propensity scores were calculated using gestational age at onset of anemia in the previous pregnancy, interval between pregnancies, number of previous births, type of alloimmunization, maternal body mass index, and number of IUTs in the previous pregnancy.

^d^≥5 SDs from fetal hemoglobin reference value (15 g/dL). 1 SD=1 g/dL difference from the reference value.

**Table S7.** IVIG Treatment Response and Associated Mortality

| Citation | Patient Group | N | | D Only and/or K Only^a^ | | Treatment (n/N [%]^b^) | Treatment Response |
| --- | --- | --- | --- | --- | --- | --- | --- |
| **IVIG Treatment Response** | | |  | |  | | |
| Ree 2020^42^ | Fetuses | 189 | | D | -- | IUT only, 183/189 (96.8)*;  IVIG+IUT, 6/189 (3.2)* | 62% decline in absolute reticulocyte count^a^ |
|  | Fetuses | 46 | | -- | K | IUT only, 44/46 (95.6)*;  IVIG+IUT, 2/46 (4.4)* | 32% decline in absolute reticulocyte count^a^ |
| Zwiers 2018^60^ | Unadjusted data, women managed with IVIG | 24 | | D or K | | IVIG+IUT, 24/24 (100);  plasmapheresis, 8/24 (33.3) | Delay of the need for IUT was on average 15 gestational days later |
|  | Weighted data,^c^ women managed with IVIG | 24 | | D or K | | IVIG+IUT, 24/24 (100);  plasmapheresis, 4/24 (15) | Delay of the need for IUT was on average 0 gestational days later |
| Bek 2019^3^ | Pregnancy | 1 | | D | -- | DFPP and IUT+IVIG, 1/1 (100) | Successful delivery at 30 weeks’ gestation |
| Colpo 2017^9^ | Pregnancy | 1 | | D | -- | TPE and IA + IVIG, 1/1 (100) | Patient required IUT because of severe fetal anemia at 30 weeks’ gestation |
| Fernandez Alba 2014^13^ | Pregnancy | 1 | | D | -- | Plasmapheresis and IVIG, 1/1 (100) | No need for IUT until delivery |
| Mayer 2018^27^ | Pregnancies | 2 | | D or K | | IVIG, 2/2 (100) | IUT was prevented in both cases |
| **IVIG-associated Mortality** | |  | |  | |  |  |
| Fox 2008^14^ | Pregnancies with prior pregnancy where fetus was hydropic and severely anemic before 20 weeks’ gestation associated with a high perinatal mortality | 6 | | D or K | | IVT±IVIG, 6/6 (100) | 1/6 (16.7)* |
|  |  | 2 | | D | -- | IVT only, 2/2 (100) | 1/2 (50)* |
|  |  | 3 | | D | -- | IVT+IVIG, 3/3 (100) | 0/3 (0) |
|  |  | 1 | | -- | K | IVT+IVIG, 1/1 (100) | 0/1 (0) |
| Kriplani 2007^21^ | Pregnancies | 4 | | D | -- | IUT+IVIG, 4/4 (100) | 0/0 (0) |
| Ruma 2007^43^ | Pregnancies, all | 9 | | D or K | | Plasmapheresis+IVIG+ IUT, 9/9 (100) | 0/0 (0) |

D, Rh(D); DFPP, double-filtration plasmapheresis; IA, immunoadsorption; IUT, intrauterine transfusion; IVIG, intravenous immunoglobulin; IVT, intravascular infusion; K, Kell; Rh, Rhesus; TPE, therapeutic plasma exchange.

^a^Data were captured only for cases with immunization types of D only, K only, or both D and K only.

^b^Values are as provided in reports, unless otherwise noted (*for values that were calculated).

^c^Data were corrected for the probability that women would be selected for IVIG treatment by their caregivers in the index pregnancy. Propensity scores were calculated using gestational age at onset of anemia in the previous pregnancy, interval between pregnancies, number of previous births, type of alloimmunization, maternal body mass index, and number of IUTs in the previous pregnancy.

**Table S8.** Overall Fetal Mortality Associated With HDFN

| Citation | Patient Group | N | D Only and/or K Only^a^ | | Treatment (n/N [%]^b^) | Mortality Rate (n/N [%]) |
| --- | --- | --- | --- | --- | --- | --- |
| Craparo 2005^10^ | Pregnancies treated with IUT | 31 | D | -- | IUT, 31/31 (100) | 4/31 (12.9)* |
| Canlorbe 2011^7^ | Pregnancies with IUT <20 weeks’ gestation | 18 | D or K | | IUT, 18/18 (100) | 3/18 (16.7)* |
| Lieberman 2020^24^ | Mothers | 16 | D | -- | No treatment, 16/16 (100) | 0/16 (0) |
|  | Mothers | 2 | -- | K | No treatment, 2/2 (100) | 0/2 (0) |
| Matijevic 2005^26^ | Pregnancies | 23 | D or K | | IUIVT, 9/13 (39.1)* (9/13* who had cordocentesis);  No IUIVT, 4/13 (60.9)* (4/13 who had cordocentesis + 10/23 who did not have cordocentesis) | 3/23 (13.0) |
| Meraj 2015^28^ | Fetuses treated with IUT | 8 | D | -- | IUT, 8/8 (100) | 1/8 (12.5)* |
| Phung 2018^32^ | Fetuses, isolated D-mediated HDFN treated with IUT | 29 | D | -- | IUT, 29/29 (100) | 2/29 (6.9) |
| Sainio 2015^44^ | Pregnancies | 94 | D or K | | IUT, 94/94 (100) | 4/94 (3.8) |
|  |  | 86 | D | -- | IUT, 86/86 (100) | 3/86 (3.5)* |
|  |  | 8 | -- | K | IUT, 8/8 (100) | 1/8 (12.5) |
| Şavkli 2020^46^ | Pregnancies treated with IUTs | 42 | D | -- | IUT, 42/42 (100) | 6/42 (14.3) |
| Somerset 2006^49^ | Anemic fetuses | 57 | D | -- | IUT, 57/57 (100) | 5/57 (8.8)* |
|  | Anemic fetuses | 7 | -- | K | IUT, 7/7 (100) | 1/7 (14.3)* |
| Tiblad 2011^53^ | Fetus (case #511) | 1 | D | -- | IUT, 1/1 (100) | 0/1 (0) |
|  | Fetus (case #512) | 1 | D | -- | IUT, 1/1 (100) | 0/1 (0) |
|  | Fetus (case #514) | 1 | D | -- | IUT, 1/1 (100) | 0/1 (0) |
|  | Fetus (case #518; 1991 pregnancy) | 1 | D | -- | IUT, 1/1 (100) | 1/1 (100) |
|  | Fetus (case #518; 1993 pregnancy) | 1 | D | -- | IUT, 1/1 (100) | 0/1 (0) |
|  | Fetus (case #249) | 1 | D | -- | IUT, 1/1 (100) | 1/1 (100) |
| van den Akker 2008^55^ | Severely hydropic fetuses | 15 | -- | K | IUT, 15/15 (100) | 2/15 (13) |
| Walsh 2013^56^ | Fetus | 11 | -- | K | IUT, 11/11 (100) | 1/11 (9.1) |
| Zwiers 2017^58^ | Fetus | 1 | D | -- | IUT, 1/1 (100); interstitial laser, 1/1 (100) | 1/1 (100)^d^ |
| Zwiers 2018^60^ | Unadjusted data, women managed with no IVIG | 28 | D or K | | IUT, 28/28 (100) | 4/28 (15) |
|  | Weighted data,^c^ women managed with no IVIG | 25 | D or K | | IUT, 25/25 (100) | 3/25 (12) |
| Fox 2008^14^ | Pregnancies with whom in a previous pregnancy the fetus had been hydropic and severely anemic prior to 20 weeks’ gestation associated with a high perinatal mortality | 5 | D or K | | IVT±IVIG, 5/5 (100) | 1/5 (20) |
|  |  | 2 | D | -- | IVT only, 2/2 (100) | 1/2 (50)* |
|  |  | 2 | D | -- | IVT+IVIG, 2/2 (100) | 0/2 (0) |
|  |  | 1 | -- | K | IVT+IVIG, 1/1 (100) | 0/1 (0) |
| Kriplani 2007^21^ | Pregnancies | 4 | D | -- | IUT+IVIG, 4/4 (100) | 0/4 (0) |
| Lakhwani 2011^22^ | Pregnancy | 1 | -- | K | Plasmapheresis+IUT, 1/1 (100) | 0/1 (0) |
| Ruma 2007^43^ | Pregnancies, all | 9 | D or K | | Plasmapheresis+ IVIG+IUT, 9/9 (100) | 0/9 (0) |
| Simonazzi 2016^48^ | Pregnancy (case # 1) | 1 | D | -- | IUT, 1/1 (100) | 1/1 (100) |

D, Rh(D); HDFN, hemolytic disease of the fetus and newborn; IUIVT, intrauterine intravascular transfusion; IUT, intrauterine transfusion; IVIG, intravenous immunoglobulin; IVT, intravascular infusion; K, Kell; Rh, Rhesus.

^a^Data were captured only for cases with immunization types of D only, K only, or both D and K only.

^b^Values are as provided in reports, unless otherwise noted (*for values that were calculated).

^c^Data were corrected for the probability that women would be selected for IVIG treatment by their caregivers in the index pregnancy. Propensity scores were calculated using gestational age at onset of anemia in the previous pregnancy, interval between pregnancies, number of previous births, type of alloimmunization, maternal body mass index, and number of IUTs in the previous pregnancy.

^d^During the study period, 39 cases of fetal or neonatal death occurred after IUT, of which 10 occurred from 2001 onward. However, number of fetal versus neonatal deaths were not reported separately.

**SUPPLEMENTAL FIGURES**

**Figure S1.** Mean Rate of Procedure-related Complications After IUT (Per Procedure [A] and Per Fetus [B]).^12,26,30,32,43,44,46,49,52,60^

CS, cesarean section; IUT, intrauterine transfusion; PROM, preterm rupture of membranes.

^a^Adverse serological outcome was defined as the development of additional maternal antibodies to anti-D and/or a ≥4-fold enhancement of antibody titer.^12^

**
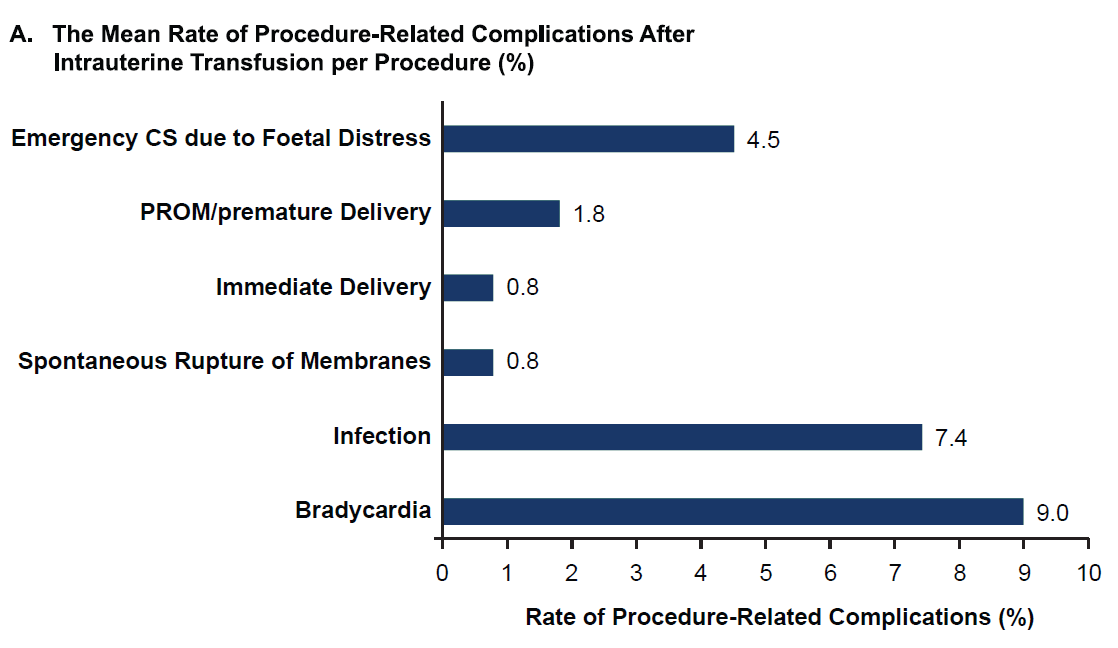
**

**
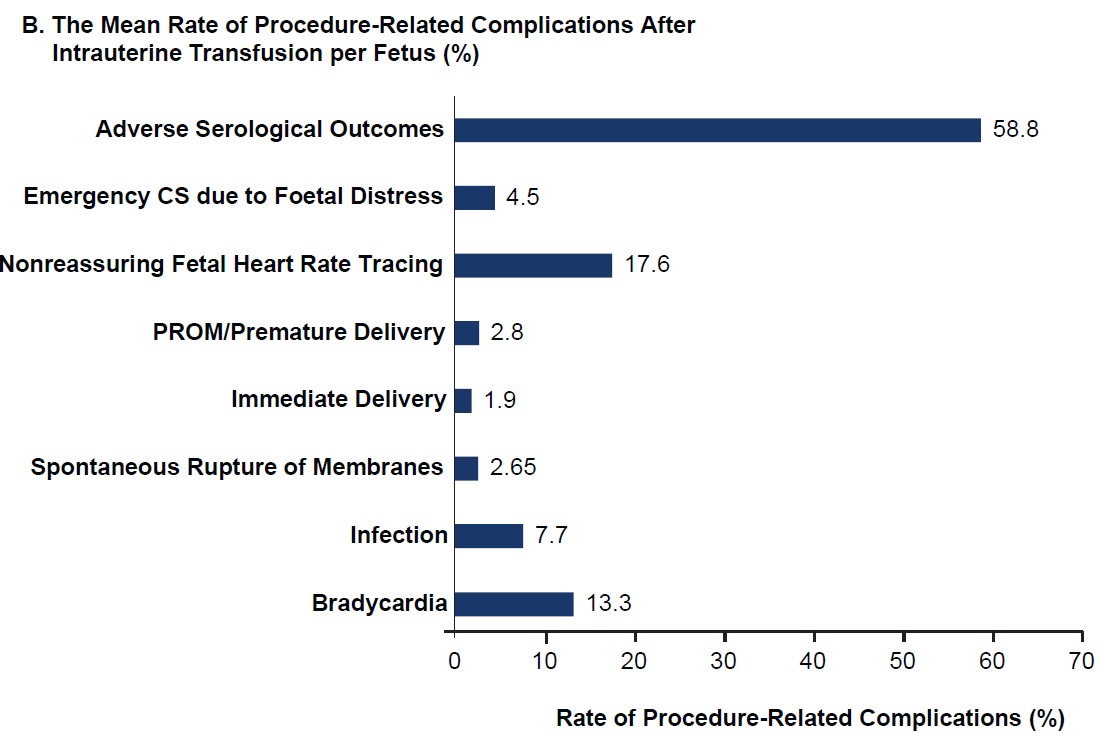
**

^a^

**Figure S2.** Flowchart of the Article Selection Process for the Humanistic and Economic Burden.

SLR, systematic literature review.

^*^From authors’ personal library.

^†^From eligible SLRs of cohort studies.


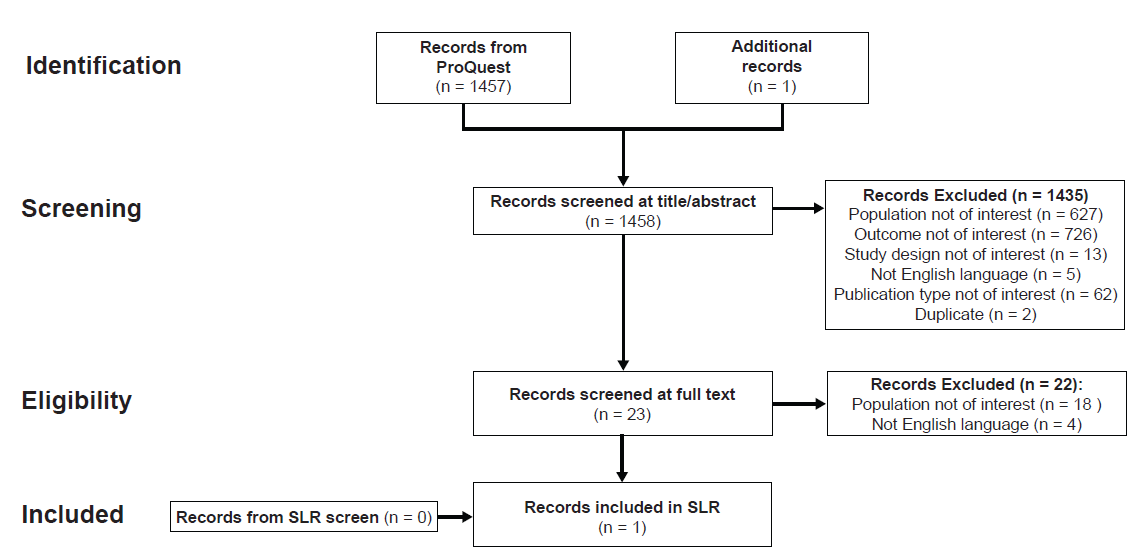


**REFERENCES**

2. Raguz MJ, Prce Z, Bjelanovic V, Bjelanovic I, Dzida S, Mabic M. 20 years of follow-up alloimmunization and hemolytic disease in newborn: has anything changed in the field over the years? Klin Padiatr. 2020;232(6):314-20.

16. Brooke BS, Schwartz TA, Pawlik TM. MOOSE reporting guidelines for meta-analyses of observational studies. JAMA Surgery. 2021;156(8):787-8.

22. Akdağ A, Erdeve O, Uraş N, Simşek Y, Dilmen U. Hydrops fetalis due to Kell alloimmunization: a perinatal approach to a rare case. Turk J Haematol. 2012;29(1):72-5.

23. Al-Dughaishi T, Al Harrasi Y, Al-Duhli M, Al-Rubkhi I, Al-Riyami N, Al Riyami A, et al. Red cell alloimmunization to Rhesus antigen among pregnant women attending a tertiary care hospital in Oman. Oman Med J. 2016;31(1):77-80.

24. Bek SG, Eren N, Uzay A, Bakirdogen S. Rh (D) alloimmunization treated by double filtration plasmapheresis. Transfus Apher Sci. 2019;58(1):83-6.

25. Bennardello F, Curciarello G. Survey on the prevention and incidence of haemolytic disease of the newborn in Italy. Blood Transfus. 2013;11(4):518-27.

26. Bi SH, Jiang LL, Dai LY, Zheng H, Zhang J, Wang LL, et al. Rh-incompatible hemolytic disease of the newborn in Hefei. World J Clin Cases. 2019;7(20):3202-7.

27. Brumbaugh JE, Morgan S, Beck JC, Zantek N, Kearney S, Bendel CM, et al. Blueberry muffin rash, hyperbilirubinemia, and hypoglycemia: a case of hemolytic disease of the fetus and newborn due to anti-Kpa. J Perinatol. 2011;31(5):373-6.

28. Canlorbe G, Mace G, Cortey A, Cynober E, Castaigne V, Larsen M, et al. Management of very early fetal anemia resulting from red-cell alloimmunization before 20 weeks of gestation. Obstet Gynecol. 2011;118(6):1323-9.

29. Chatziantoniou V, Heeney N, Maggs T, Rozette C, Fountain C, Watts T, et al. A descriptive single-centre experience of the management and outcome of maternal alloantibodies in pregnancy. Transfus Med. 2017;27(4):275-85.

30. Colpo A, Tison T, Gervasi MT, Vio C, Vicarioto M, De Silvestro G, et al. Personalized treatment with immunoadsorption and intravenous immunoglobulin in a case of severe Rh alloimmunization during pregnancy unresponsive to plasma - exchange. Transfus Apher Sci. 2017;56(3):480-3.

31. Craparo FJ, Bonati F, Gementi P, Nicolini U. The effects of serial intravascular transfusions in ascitic/hydropic RhD-alloimmunized fetuses. Ultrasound Obstet Gynecol. 2005;25(2):144-8.

32. de Assunção RA, Liao AW, Brizot Mde L, Francisco RP, Zugaib M. Changes in fetal myocardial performance index following intravascular transfusion: preliminary report. J Matern Fetal Neonatal Med. 2016;29(16):2697-702.

33. Dubey A, Sonker A, Chaudhary R. Enhancement of antibody titre and development of additional red cell alloantibodies following intrauterine transfusion. Indian J Hematol Blood Transfus. 2016;32(1):92-4.

34. Fernández Alba JJ, León R, González-Macías C, Paz A, Prado F, Moreno LJ, et al. Treatment of D alloimmunization in pregnancy with plasmapheresis and intravenous immune globulin: case report. Transfus Apher Sci. 2014;51(1):70-2.

35. Fox C, Martin W, Somerset DA, Thompson PJ, Kilby MD. Early intraperitoneal transfusion and adjuvant maternal immunoglobulin therapy in the treatment of severe red cell alloimmunization prior to fetal intravascular transfusion. Fetal Diagn Ther. 2008;23(2):159-63.

36. Gottvall T, Filbey D. Alloimmunization in pregnancy during the years 1992-2005 in the central west region of Sweden. Acta Obstet Gynecol Scand. 2008;87(8):843-8.

37. Gudlaugsson B, Hjartardottir H, Svansdottir G, Gudmundsdottir G, Kjartansson S, Jonsson T, et al. Rhesus D alloimmunization in pregnancy from 1996 to 2015 in Iceland: a nation-wide population study prior to routine antenatal anti-D prophylaxis. Transfusion. 2020;60(1):175-83.

38. Haider M, Memon S, Tariq F, Fatima S, Hameed A. Rhesus isoimmunization: late-onset hemolytic disease of the newborn without jaundice. Cureus. 2020;12(1):e6559.

39. Harper DC, Swingle HM, Weiner CP, Bonthius DJ, Aylward GP, Widness JA. Long-term neurodevelopmental outcome and brain volume after treatment for hydrops fetalis by in utero intravascular transfusion. Am J Obstet Gynecol. 2006;195(1):192-200.

40. Hassan MZ, Iberahim S, Abdul Rahman WSW, Zulkafli Z, Bahar R, Ramli M, et al. Severe anti-D haemolytic disease of fetal and newborn in rhesus D negative primigravida. Malays J Pathol. 2019;41(1):55-8.

41. Karagol BS, Zenciroglu A, Okumus N, Karadag N, Dursun A, Hakan N. Hemolytic disease of the newborn caused by irregular blood subgroup (Kell, C, c, E, and e) incompatibilities: report of 106 cases at a tertiary-care centre. Am J Perinatol. 2012;29(6):449-54.

42. Kriplani A, Malhotra Singh B, Mandal K. Fetal intravenous immunoglobulin therapy in Rhesus hemolytic disease. Gynecol Obstet Invest. 2007;63(3):176-80.

43. Lakhwani S, Machado P, Pecos P, Coloma M, Rebollo S, Raya JM. Kell hemolytic disease of the fetus. Combination treatment with plasmapheresis and intrauterine blood transfusion. Transfus Apher Sci. 2011;45(1):9-11.

44. Levy-Zauberman Y, Mailloux A, Kane A, Castaigne V, Cortey A, Carbonne B. Massive fetomaternal hemorrhage secondary to intrauterine intravascular transfusion. Obstet Gynecol. 2011;118(2 Pt 2):439-42.

45. Lieberman L, Callum J, Cohen R, Cserti-Gazdewich C, Ladhani NNN, Buckstein J, et al. Impact of red blood cell alloimmunization on fetal and neonatal outcomes: a single center cohort study. Transfusion. 2020;60(11):2537-46.

46. Manoura A, Korakaki E, Hatzidaki E, Saitakis E, Maraka S, Papamastoraki I, et al. Use of recombinant erythropoietin for the management of severe hemolytic disease of the newborn of a K0 phenotype mother. Pediatr Hematol Oncol. 2007;24(1):69-73.

47. Matijevic R, Grgic O, Klobucar A, Miskovic B. Diagnosis and management of Rh alloimmunization. Fetal Diagn Ther. 2005;20(5):393-401.

48. Mayer B, Hinkson L, Hillebrand W, Henrich W, Salama A. Efficacy of antenatal intravenous immunoglobulin treatment in pregnancies at high risk due to alloimmunization to red blood cells. Transfus Med Hemother. 2018;45(6):429-36.

49. Meraj B, Mobusher I, Waheed S, Waseem M, Rashid Y. Role of intrauterine blood transfusions in management of Rh-isoimmunized pregnancies. Pakistan J Medical Health Sci. 2015;9:318-21.

50. Navarro M, Negre S, Matoses ML, Golombek SG, Vento M. Necrotizing enterocolitis following the use of intravenous immunoglobulin for haemolytic disease of the newborn. Acta Paediatr. 2009;98(7):1214-7.

51. Nwogu LC, Moise KJ Jr, Klein KL, Tint H, Castillo B, Bai Y. Successful management of severe red blood cell alloimmunization in pregnancy with a combination of therapeutic plasma exchange, intravenous immune globulin, and intrauterine transfusion. Transfusion. 2018;58(3):677-84.

52. Palfi M, Hildén JO, Matthiesen L, Selbing A, Berlin G. A case of severe Rh (D) alloimmunization treated by intensive plasma exchange and high-dose intravenous immunoglobulin. Transfus Apher Sci. 2006;35(2):131-6.

53. Phung TV, Houfflin-Debarge V, Ramdane N, Ghesquière L, Delsalle A, Coulon C, et al. Maternal red blood cell alloimmunization requiring intrauterine transfusion: a comparative study on management and outcome depending on the type of antibody. Transfusion. 2018;58(5):1199-205.

54. Rahimi-Levene N, Chezar J, Yahalom V. Red blood cell alloimmunization prevalence and hemolytic disease of the fetus and newborn in Israel: A retrospective study. Transfusion. 2020;60(11):2684-90.

55. Rahimi-Sharbaf F, Niromanesh S, Talebzadeh Z, Kaveh M, Nayary F. Rh alloimmunization and term delivery. Arch Iran Med. 2007;10(1):111-3.

56. Rath ME, Smits-Wintjens VE, Lindenburg IT, Brand A, van Kamp IL, Oepkes D, et al. Exchange transfusions and top-up transfusions in neonates with Kell haemolytic disease compared to Rh D haemolytic disease. Vox Sang. 2011;100(3):312-6.

57. Rath ME, Smits-Wintjens VE, Oepkes D, van Zwet EW, van Kamp IL, Brand A, et al. Thrombocytopenia at birth in neonates with red cell alloimmune haemolytic disease. Vox Sang. 2012;102(3):228-33.

58. Rath ME, Smits-Wintjens VE, Oepkes D, Walther FJ, Lopriore E. Iron status in infants with alloimmune haemolytic disease in the first three months of life. Vox Sang. 2013;105(4):328-33.

59. Rath ME, Smits-Wintjens VE, Lindenburg IT, Folman CC, Brand A, van Kamp IL, et al. Postnatal outcome in neonates with severe Rhesus c compared to Rhesus D hemolytic disease. Transfusion. 2013;53(7):1580-5.

60. Ree IMC, de Haas M, Middelburg RA, Zwiers C, Oepkes D, van der Bom JG, et al. Predicting anaemia and transfusion dependency in severe alloimmune haemolytic disease of the fetus and newborn in the first 3 months after birth. Br J Haematol. 2019;186(4):565-73.

61. Ree IMC, Lopriore E, Zwiers C, Böhringer S, Janssen MWM, Oepkes D, et al. Suppression of compensatory erythropoiesis in hemolytic disease of the fetus and newborn due to intrauterine transfusions. Am J Obstet Gynecol. 2020;223(1):119.e1-10.

62. Ree IMC, de Grauw AM, Bekker V, de Haas M, Te Pas AB, Oepkes D, et al. Necrotizing enterocolitis in haemolytic disease of the newborn: a retrospective cohort study. Vox Sang. 2020;115(2):196-201.

63. Ruma MS, Moise KJ Jr, Kim E, Murtha AP, Prutsman WJ, Hassan SS, et al. Combined plasmapheresis and intravenous immune globulin for the treatment of severe maternal red cell alloimmunization. Am J Obstet Gynecol. 2007;196(2):138.e1-6.

64. Sainio S, Nupponen I, Kuosmanen M, Aitokallio-Tallberg A, Ekholm E, Halmesmäki E, et al. Diagnosis and treatment of severe hemolytic disease of the fetus and newborn: a 10-year nationwide retrospective study. Acta Obstet Gynecol Scand. 2015;94(4):383-90.

65. Santos MC, Sá C, Gomes SC Jr, Camacho LA, Moreira ME. The efficacy of the use of intravenous human immunoglobulin in Brazilian newborns with rhesus hemolytic disease: a randomized double-blind trial. Transfusion. 2013;53(4):777-82.

66. Şavkli AÖ, Çetin BA, Acar Z, Özköse Z, Behram M, Çaypinar SS, et al. Perinatal outcomes of intrauterine transfusion for foetal anaemia due to red blood cell alloimmunisation. J Obstet Gynaecol. 2020;40(5):649-53.

67. Sikkel E, Klumper FJ, Oepkes D, Teunissen AK, Meerman RH, Le Cessie S, et al. Fetal cardiac contractility before and after intrauterine transfusion. Ultrasound Obstet Gynecol. 2005;26(6):611-7.

68. Simonazzi G, Bernabini D, Curti A, Bisulli M, Pilu G, Brill CB, et al. Fetal cerebellar damage in fetuses with severe anemia undergoing intrauterine transfusions. J Matern Fetal Neonatal Med. 2016;29(3):389-92.

69. Somerset DA, Moore A, Whittle MJ, Martin W, Kilby MD. An audit of outcome in intravascular transfusions using the intrahepatic portion of the fetal umbilical vein compared to cordocentesis. Fetal Diagn Ther. 2006;21(3):272-6.

70. Takcı S, Alarcon-Martinez T, Bozkaya D, Yiğit Ş, Korkmaz A, Yurdakök M. Cholestasis in infants with immune hydrops fetalis. Turk J Pediatr. 2013;55(6):616-9.

71. Tara F, Maleki A, Taheri N, Moein Darbari S. A case of D alloimmunization in pregnancy: successfully treated solely with therapeutic plasma exchange (TPE). J Blood Med. 2019;10:251-3.

72. Temel Yüksel İ, Acar D, Turhan U, Aslan Çetİn B, Köroğlu N, Şenol G, et al. Assessment of fetal right ventricular myocardial performance index changes following intrauterine transfusion. J Matern Fetal Neonatal Med. 2021;34(18):3046-9.

73. Tiblad E, Kublickas M, Ajne G, Bui TH, Ek S, Karlsson A, et al. Procedure-related complications and perinatal outcome after intrauterine transfusions in red cell alloimmunization in Stockholm. Fetal Diagn Ther. 2011;30(4):266-73.

74. Urutherakumar V, Welsh A, Henry A. Short-term outcomes following intrauterine transfusions for fetal anaemia: a retrospective cohort study. Aust N Z J Obstet Gynaecol. 2020;60(5):738-45.

75. van den Akker ES, Klumper FJ, Brand A, Kanhai HH, Oepkes D. Kell alloimmunization in pregnancy: associated with fetal thrombocytopenia? Vox Sang. 2008;95(1):66-9.

76. Walsh CA, Russell N, McAuliffe FM, Higgins S, Mahony R, Carroll S, et al. Relationship between maternal antibody type and antenatal course following intrauterine transfusion for red cell alloimmunisation. Eur J Obstet Gynecol Reprod Biol. 2013;171(2):235-9.

77. Xu W. A case of severe Rh (D) alloimmunization pregnant woman delivery an infant with limited treatment. Transfus Apher Sci. 2013;49(2):168-70.

78. Zwiers C, Lindenburg ITM, Klumper FJ, de Haas M, Oepkes D, Van Kamp IL. Complications of intrauterine intravascular blood transfusion: lessons learned after 1678 procedures. Ultrasound Obstet Gynecol. 2017;50(2):180-6.

79. Zwiers C, Oepkes D, Lopriore E, Klumper FJ, de Haas M, van Kamp IL. The near disappearance of fetal hydrops in relation to current state-of-the-art management of red cell alloimmunization. Prenat Diagn. 2018;38(12):943-50.

80. Zwiers C, van der Bom JG, van Kamp IL, van Geloven N, Lopriore E, Smoleniec J, et al. Postponing early intrauterine transfusion with intravenous immunoglobulin treatment; the PETIT study on severe hemolytic disease of the fetus and newborn. Am J Obstet Gynecol. 2018;219(3):291.e1-9.
