# Supplementary material for: Hemolytic disease of the fetus and newborn: systematic literature review of the antenatal landscape
Source: BMC Pregnancy Childbirth. 2023 Jan 7;23:12. doi: 10.1186/s12884-022-05329-z (PMC9824959; doi:10.1186/s12884-022-05329-z)
Supplement: Supplementary file 2 — Additional file 2: Appendix S1. Search Strategy. Appendix S2. Search Strategy for the Humanistic and Economic Burden. [file 12884_2022_5329_MOESM2_ESM.pdf]

Appendix S1. Search Strategy

#	Description	Search String	Hits
S1	Disease state	(EMB.EXACT.EXPLODE("newborn hemolytic disease") OR MESH.EXACT.EXPLODE("Erythroblastosis, Fetal")) AND (TI,AB(HDFN OR ((hemolytic OR haemolytic) NEAR/3 (fetus OR newborn OR foetus)))) OR TI,AB(“hemolytic disease of the fetus and newborn” OR “haemolytic disease of the fetus and newborn” OR “hemolytic disease of the foetus and newborn” OR “haemolytic disease of the foetus and newborn” OR “hemolytic disease of the newborn” OR “haemolytic disease of the newborn” OR HDFN) OR EMB.EXACT.EXPLODE("alloimmunization") OR (“maternal alloimmunization”) OR (“red cell alloimmunization”)	12,499[a]
S2	Intervention/treatment setting	EMB.EXACT("intrauterine blood transfusion") OR EMB.EXACT("fetal therapy") OR EMB.EXACT.EXPLODE("neonatal intensive care unit") OR MESH.EXACT.EXPLODE("Fetal Therapies") OR MESH.EXACT("Intensive Care Units, Neonatal") OR EMB.EXACT("immunoglobulin") OR MESH.EXACT("Immunoglobulins, Intravenous") OR EMB.EXACT.EXPLODE("plasma exchange") OR MESH.EXACT.EXPLODE("Plasma Exchange") OR (“therapeutic plasma exchange”) OR (“neonatal transfusion”) OR EMB.EXACT.EXPLODE("exchange blood transfusion") OR (“neonatal exchange transfusion”)	233,001[a]

S3	Clinical outcomes	MESH.EXACT("Mortality") OR EMB.EXACT("mortality") OR EMB.EXACT("infant mortality") OR MESH.EXACT.EXPLODE("Infant Mortality") OR MESH.EXACT(Morbidity) OR EMB.EXACT(morbidity) OR MESH.EXACT.EXPLODE("Anemia, Neonatal") OR MESH.EXACT.EXPLODE("Hyperbilirubinemia") OR MESH.EXACT("Fetal Blood") OR MESH.EXACT("Perinatal Death") OR MESH.EXACT("Fetal Death") OR EMB.EXACT("newborn jaundice") OR EMB.EXACT("neonatal hyperbilirubinemia") OR EMB.EXACT("anemia") OR EMB.EXACT("hyperbilirubinemia") OR EMB.EXACT.EXPLODE("perinatal morbidity") OR EMB.EXACT("newborn death") OR EMB.EXACT("fetus death") OR EMB.EXACT("rhesus incompatibility") OR (late NEAR/3 anemia) OR TI,AB(complication*) OR TI,AB(adverse /NEAR1 (effect* OR reaction* OR event*)) OR MESH.EXACT("Hydrops Fetalis") OR EMB.EXACT.EXPLODE("edema") OR EMB.EXACT.EXPLODE("fetus hydrops") OR MESH.EXACT.EXPLODE("Premature Birth") OR EMB.EXACT.EXPLODE("prematurity") OR (emergency NEAR/2 childbirth OR delivery OR cesarean section) OR MESH.EXACT.EXPLODE("Respiration, Artificial") OR EMB.EXACT("artificial ventilation") OR	6,724,924[a]

		MESH.EXACT("Heart Failure") OR EMB.EXACT("heart failure")	
S4	Combined disease state and outcomes of interest	S1 AND S2	1,903[b]
S5	Combined disease state and outcomes of interest	S1 AND S3	3,619[b]
S6	Total hits	S4 OR S5	5,069[a]
S7	Final hits published between January 1, 2005, and March 10, 2021	S6 AND publication date (>2004)	2,518[b]

[a]Duplicates were removed from the search but included in the result count.

[b]Duplicates were removed from the search and from the result count.

Appendix S2. Search Strategy for the Humanistic and Economic Burden

#	Description	Search String	Hits
S1	Disease state	(EMB.EXACT.EXPLODE("newborn hemolytic disease") OR MESH.EXACT.EXPLODE("Erythroblastosis, Fetal")) AND (TI,AB(HDFN OR ((hemolytic OR haemolytic) NEAR/3 (fetus OR newborn OR foetus)))) OR TI,AB(“hemolytic disease of the fetus and newborn” OR “haemolytic disease of the fetus and newborn” OR “hemolytic disease of the foetus and newborn” OR “haemolytic disease of the foetus and newborn” OR “hemolytic disease of the newborn” OR “haemolytic disease of the newborn” OR HDFN) OR EMB.EXACT.EXPLODE("alloimmunization") OR (“maternal alloimmunization”) OR (“red cell alloimmunization”)	12499*
S2	Intervention/ Tx setting	EMB.EXACT("intrauterine blood transfusion") OR EMB.EXACT("fetal therapy") OR EMB.EXACT.EXPLODE("neonatal intensive care unit") OR MESH.EXACT.EXPLODE("Fetal Therapies") OR MESH.EXACT("Intensive Care Units, Neonatal") OR EMB.EXACT("immunoglobulin") OR MESH.EXACT("Immunoglobulins, Intravenous") OR EMB.EXACT.EXPLODE("plasma exchange") OR MESH.EXACT.EXPLODE("Plasma Exchange") OR (“therapeutic plasma exchange”) OR (“neonatal transfusion”) OR EMB.EXACT.EXPLODE("exchange blood transfusion") OR (“neonatal exchange transfusion”)	233001*

S3	Humanistic Outcomes	EMB.EXACT("International Classification of Functioning, Disability and Health" OR "quality of life" OR "short form 36" OR "patient reported outcome" OR "patient preference" OR "questionnaire" OR "quality adjusted life years") OR EMB.EXACT.EXPLODE("health status indicator") OR MESH.EXACT("International Classification of Functioning, Disability and Health" OR "Quality of Life" OR "Value of Life" OR "Patient Reported Outcome Measures" OR "Patient Preference" OR Questionnaires OR "Quality-Adjusted Life Years") OR MESH.EXACT.EXPLODE("Health Status Indicators") OR TI,AB(burden OR (impact NEAR/3 (caregiver OR family OR families OR society OR societal or patient OR person)) OR "unmet need" OR "disability adjusted" OR DALY* OR "dartmouth coop" OR "Duke health profile" OR EQ OR (EURO NEAR/2 (QUAL OR QOL)) OR ((daily OR day) NEAR/3 activit*) OR "functional status" OR FSQ OR (function* NEAR/5 (reduc* OR impair* OR decrease* OR impact*)) OR "quality of life" OR QOL OR hrqol OR hrql OR hql OR hqol OR "hr qol" OR "h qol" OR "life quality" OR (health NEAR/3 (status OR indicator*)) OR "Nottingham health" OR NHP OR PQOL OR "perceived quality" OR QLS OR "quality of life scale" OR wellbeing OR "well being" OR QWB OR rosser OR SF OR "short form" OR "shortform*" OR "sickness impact" OR SIP OR "patient reported" OR "self reported" OR QALY* OR QALD* OR QALE* OR QTIME* OR (utilit* NEAR/3 (valu* OR measur* OR	4018894*

		health OR life OR estimat* OR elicit* OR disease OR score* OR weight OR instrument OR instruments OR index)) OR “quality adjusted” OR “life year*” OR “health year*” OR disutilit* OR “willingness to pay” OR WTP OR (preference* NEAR/3 (valu* OR measur* OR health OR life OR estimat* OR elicit* OR disease OR score* OR instrument OR instruments OR index)) OR “healthy utility index” OR hui OR “standard gamble” OR “time trade off” OR “time tradeoff” OR TTO OR “health assessment questionnaire” OR “health assessment questionnaires” OR HAQ OR (humanistic AND burden))	
S4	Economic Outcomes	MESH.EXACT(“Cost of Illness” OR “Economics, Hospital” OR “Economics, Nursing” OR “Economics, Pharmaceutical” OR “Fees and Charges” OR “Economics, Dental” OR “Employer Health Costs” OR “Efficiency” OR “Presenteeism” OR “Absenteeism”) OR MESH.EXACT.EXPLODE(“Health Care Costs” OR “Health Expenditures” OR “Economics, Medical” OR “Salaries and Fringe Benefits”) OR EMB.EXACT(“cost of illness” OR “drug cost” OR “productivity” OR “medical leave” OR “presenteeism” OR “absenteeism”) OR EMB.EXACT.EXPLODE(“health care cost” OR “salary and fringe benefit”) OR TI,AB(presenteeism OR absenteeism OR (cost* NEAR/3 (medical OR direct OR indirect OR drug OR pharmaceutical OR hospital OR emergency OR outpatient OR inpatient OR ambulatory OR “primary care” OR practitioner OR device OR informal OR economic OR societal OR	2205185*

		intangible OR caregiver OR physician OR specialist OR healthcare OR “health care” OR annual* OR clinic)) OR ((burden OR impact) NEAR/3 (cost OR costs OR economic OR economics OR caregiver OR caregivers OR family OR families OR society OR societal OR employee OR employer)) OR (los* AND work AND day*) OR “sick day” OR “sick leave” OR “sickness absence” OR “work absence” OR “work incapacity” OR “work leave” OR “disability absence” OR ((resource OR healthcare OR “health care”) NEAR/5 (use OR utilization OR utilisation)) OR ((visit* OR admission* OR readmission* OR stay* OR day*) NEAR/3 (physician OR emergency OR specialist OR outpatient OR inpatient OR “primary care” OR practitioner OR hospital OR clinic)) OR hospitalization OR hospitalisation OR “length of stay” OR “LOS” OR (burden AND financial))	
S5	Combined disease state and outcomes of interest	S1 AND S2	1903°
S6		S1 AND S3	329°
S7		S1 AND S4	344°
S8	Total Hits	S5 OR S6 OR S7	2387°
S9	Final hits published on or after 2005	S8 and PD(>2004)	1441°

* Duplicates are removed from the search but included in the result count.

° Duplicates are removed from the search and from the result count.
